# Rapid affinity chromatographic isolation method for LDL in human plasma by immobilized chondroitin-6-sulfate and anti-apoB-100 antibody monolithic disks in tandem

**DOI:** 10.1038/s41598-019-47750-z

**Published:** 2019-08-02

**Authors:** Thanaporn Liangsupree, Evgen Multia, Jari Metso, Matti Jauhiainen, Patrik Forssén, Torgny Fornstedt, Katariina Öörni, Aleš Podgornik, Marja-Liisa Riekkola

**Affiliations:** 1 Department of Chemistry, University of Helsinki, P.O. Box 55, FI-00014 Helsinki, Finland; 2 Minerva Foundation Institute for Medical Research and National Institute for Health and Welfare, Biomedicum 2U, Helsinki, Finland; 3 Department of Engineering and Chemical Sciences, Karlstad University, SE-651 88 Karlstad, Sweden; 4 Wihuri Research Institute, Atherosclerosis Research Laboratory, Haartmaninkatu 8, 00290 Helsinki, Finland; 5 Faculty of Chemistry and Chemical Technology, University of Ljubljana, Ljubljana, Slovenia; 6 Center of Excellence for Biosensors, Instrumentation and Process Control - COBIK, Ajdovščina, Slovenia

**Keywords:** Cardiology, Biophysical methods

## Abstract

Low-density lipoprotein (LDL) is considered the major risk factor for the development of atherosclerotic cardiovascular diseases (ASCVDs). A novel and rapid method for the isolation of LDL from human plasma was developed utilising affinity chromatography with monolithic stationary supports. The isolation method consisted of two polymeric monolithic disk columns, one immobilized with chondroitin-6-sulfate (C6S) and the other with apolipoprotein B-100 monoclonal antibody (anti-apoB-100 mAb). The first disk with C6S was targeted to remove chylomicrons, very-low-density lipoprotein (VLDL) particles, and their remnants including intermediate-density lipoprotein (IDL) particles, thus allowing the remaining major lipoprotein species, i.e. LDL, lipoprotein(a) (Lp(a)), and high-density lipoprotein (HDL) to flow to the anti-apoB-100 disk. The second disk captured LDL particles via the anti-apoB-100 mAb attached on the disk surface in a highly specific manner, permitting the selective LDL isolation. The success of LDL isolation was confirmed by different techniques including quartz crystal microbalance. In addition, the method developed gave comparable results with ultracentrifugation, conventionally used as a standard method. The reliable results achieved together with a short isolation time (less than 30 min) suggest the method to be suitable for clinically relevant LDL functional assays.

## Introduction

Low-density lipoprotein (LDL) is a major cholesterol carrier in circulation. LDL is associated with the initiation and progression of atherosclerotic cardiovascular diseases due to particle remodeling and its retention within the subendothelial matrix and accumulation in subendothelial macrophages^1,2^. The major apolipoprotein of the LDL particles, apolipoprotein B-100 (apoB-100), interacts tightly with the components of the extracellular matrix of the arterial intima thus enhancing its accumulation. In addition to LDL, the triglyceride-rich lipoproteins, including chylomicrons and VLDL remnants, are also capable of penetrating the arterial intima thus promoting atherogenesis^3–5^. Rapid isolation of LDL particles from human plasma is crucially needed for their functional, lipidomic, and proteomic studies. Currently, the most employed method for the LDL isolation is based on ultracentrifugation^6,7^. Major disadvantages of this method include being time-consuming, laborious, and prone to contamination with other lipoproteins^8,9^. Therefore, a new and faster method that permits higher selectivity and specificity is needed to obtain LDL with high purity.

Immunoaffinity chromatography (IAC) utilising specific interactions between an affinity ligand, that is typically a monospecific antibody, and its corresponding target antigen, is amongst the most selective methods for the isolation of specific biomolecules^10,11^. IAC has been extensively used to isolate lipoprotein species from plasma and then LDL particles are traditionally co-isolated with VLDL and other anti-apoB-containing lipoproteins using anti-apoB antibodies immobilized on crosslinked agarose^12,13^. However, although it is an applicable method, agarose matrix inherently lacks mechanical strength and suffers from swelling in aqueous solution and from slow mass transport^14^. In recent years, monolithic stationary supports in IAC have attracted more and more interest for fast isolation of low-abundant biomolecules from complex biological matrices^15–21^. Monolithic stationary phase is defined as a single piece of interconnected porous material with through-pore networks. Polymeric monoliths, such as methacrylate-based monoliths, are favourable due to their high mechanical stability, tolerance over a wide pH range, and lack of swelling. In addition, when compared to conventional particle-based supports (e.g. beads, silica particles) in which mass transport of analytes is governed by diffusion into and out of the pores^22^, it is based on convective flow through macropores in monoliths, allowing significantly faster mass transfer than diffusion. Thus, monoliths are extremely beneficial for large biomolecules with low diffusivity. Moreover, the convective flow permits operations at high flow rates without suffering from backpressure, resulting in short analysis and isolation time, while maintaining satisfactory and flow-unaffected separation efficiencies^23–28^. Therefore, the combination of IAC and polymeric monolithic supports is a promising method for rapid and selective LDL isolation.

In this study, we developed an affinity chromatographic method for fast isolation of LDL from human plasma with the total isolation time of less than 30 min. The system contained two polymeric monolithic disk columns, one immobilized with chondroitin 6-sulfate (C6S), a glycosaminoglycan capable of interacting with apoE and apoB, and the second with anti-apoB-100 mAb. The aim of the C6S disk was to isolate VLDL particles and their remnants including IDL particles from plasma so that the anti-apoB-100 mAb could specifically capture LDL particles without co-isolation of other apoB-100-containing lipoproteins. The success of LDL isolation was confirmed by a variety of techniques, including quartz crystal microbalance (QCM). The QCM biosensor was extremely beneficial for the comparison of the new isolation system to commonly used reference ultracentrifugation method.

## Results and Discussion

In this section, we present the development of a new affinity chromatographic method for a quick isolation of LDL particles from plasma, followed by several techniques including QCM by utilising Adaptive Interaction Distribution Algorithm (AIDA)^29^ to confirm the reliability of our method.

### Development of the isolation method

#### Selection of monolithic stationary phase and affinity ligands

In this study, we selected poly(glycidyl methacrylate-*co*-ethylene dimethacrylate) (poly(GMA-*co*-EDMA)) monolithic disk columns activated with carbonyldiimidazole (CDI) as stationary supports. The polymeric monolith permits the use of a wide pH range needed for analyte desorption due to its mechanical stability. The interconnected channels (pore size of 1.3 µm) allow all plasma lipoproteins (radius up to 1.2 µm), including chylomicrons, VLDL, VLDL remnants, including IDL, Lp(a), LDL, and HDL, to flow through the channels to their binding sites^30^. In addition, the imidazole carbamate group on the monolith surface can be directly coupled with affinity ligands containing amines, e.g. antibody, via nucleophilic substitution to yield a stable carbamate linkage^26^. This immobilization method is faster and requires fewer steps compared to other methods, such as epoxy method^31,32^.

**Figure 1:**
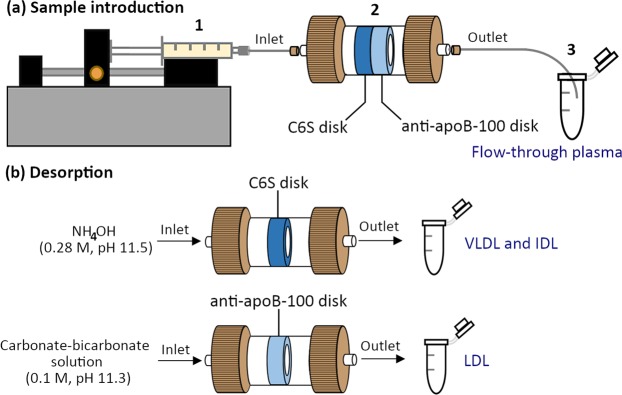
(**a**) Schematic illustration of LDL isolation system consisting of tandemly connected C6S and anti-apoB-100 disks: (1) introduction of plasma sample using a syringe pump, (2) two monolithic disk columns immobilized with C6S and anti-apoB-100 mAb in a housing cartridge, and (3) collection of the flow-through plasma. (**b**) Separate desorption of the C6S disk bound molecules with NH_4_OH (0.28 M, pH 11.5) (top) and anti-apoB-100 disk molecules with carbonate-bicarbonate solution (0.1 M, pH 11.3) (bottom). VLDL and IDL were collected from the C6S disk, while LDL was desorbed specifically from the anti-apoB-100 disk.

Next, we selected suitable affinity ligands based on their binding characteristics to the lipoprotein particles. QCM and a new numerical algorithm, AIDA^29^, were exploited to study the interactions between C6S and an ultracentrifugally purified mixture of chylomicrons, VLDL particles, VLDL remnants, including IDL particles (namely VLDL-IDL mixture), and between the anti-apoB-100 mAb and LDL particles. C6S is a class of unbranched glycosaminoglycans, which show affinity towards proteins having positively charged segments, such as apolipoproteins E and B, through electrostatic interactions between the negatively-charged groups of C6S chains and the positively-charged groups at the apolipoprotein polypeptide chain^33,34^. C6S was selected for our study to capture lipoprotein classes containing apoE and apoB, i.e., chylomicrons, VLDL particles, and their remnants including IDL particles^35,36^. The QCM studies of the interaction between C6S and the VLDL-IDL mixture showed slow to negligible dissociation of these lipoprotein particles from the C6S ligand (Supplementary Fig. S1). This verified the ability of C6S to strongly retain these lipoprotein particles. In addition to the C6S ligand, as our previous study^37^ demonstrated, the anti-apoB-100 mAb displayed strong and specific affinity towards anti-apoB-100-containing lipoproteins, i.e., VLDL, IDL, and LDL, and it was used as the immunoaffinity ligand for LDL isolation in this study. The isolation system was designed in such a way that the C6S and the anti-apoB-100 disks were arranged in series in a housing cartridge with the C6S disk being the first disk that contacts with plasma as illustrated in Fig. 1a. The C6S disk aims to capture VLDL particles and possible VLDL remnants, but preferably not LDL particles, to prevent them from reaching the anti-apoB-100 disk, while allowing the LDL particles to continue their flow to the anti-apoB-100 disk (Fig. 1a).

#### Selection of eluents

**Figure 2:**
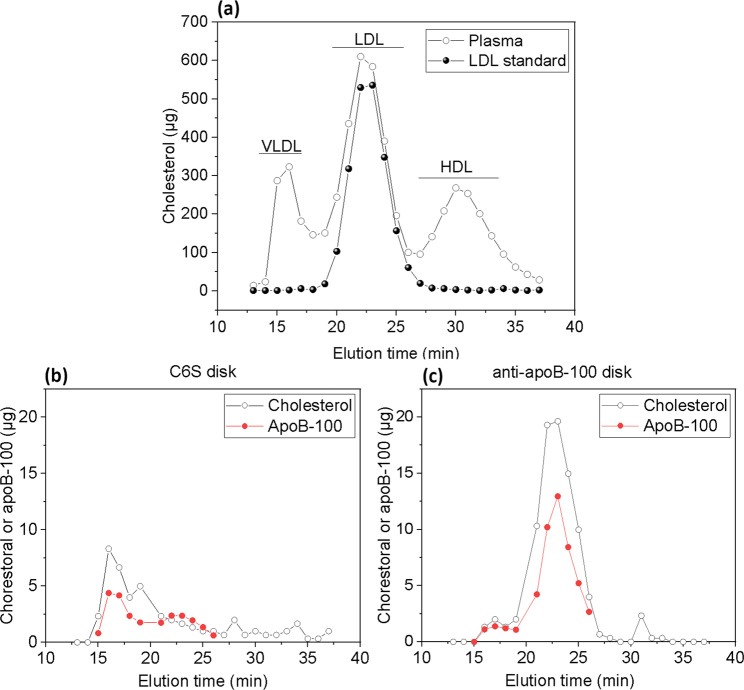
Lipoprotein profiles of human plasma and ultracentrifugally isolated LDL using FPLC-size exclusion chromatography analysis. Panel (a) cholesterol contents in eluted plasma (320 µL) lipoprotein (VLDL, LDL, HDL) fractions. Cholesterol profile of ultracentrifugally isolated LDL (500 µL, normalized to 1 mg of LDL protein) is also displayed. Panel (b) cholesterol and apoB-100 levels of the FPLC-isolated fractions from the C6S disk, and from the anti-apoB-100 disk, panel (c). Flow rate in FPLC analysis was 0.5 mL/min and 0.5 mL fractions were collected for lipid and protein analyses. The plasma samples injected to the C6S and anti-apoB-100 disk system were 320 µL diluted to 5 mL with PBS (Duplicate of 320 µL).

For the further elucidation of plasma and lipoprotein samples, it was essential to fractionate all samples using fast protein liquid chromatography (FPLC)-size exclusion chromatography (SEC). The FPLC-SEC system fractionated lipoproteins based on their sizes, allowing a primary detection of proteins and lipoprotein subclasses. Then, the fractions were subjected to further cholesterol and protein analyses (Fig. S2). The cholesterol profiles of fractionated non-fasting plasma in Fig. 2a verify cholesterol-rich fractions at the elution position of LDL. The primary detection and fractionation of the lipoproteins were performed in throughout the study to further analyse the samples.

After the immobilization of affinity ligands to the monolithic disks, we first studied the effect of different eluents on affinity chromatographic method using C6S and anti-apoB-100 disks. Different pH and ionic strength conditions (Supplementary Tables S1 and S2) were tested by using the VLDL-IDL mixture for the C6S disk and LDL for the anti-apoB-100 disk. First, the VLDL-IDL mixture was injected to the C6S disk, and then LDL was injected to the anti-apoB-100 disk. Among the tested eluents, to our surprise, only eluents at alkaline pH (pH 11.3 and 11.5) were capable of releasing bound particles from both disks as demonstrated in Supplementary Figs S3 and S4. Then, we further investigated the anti-apoB-100 disk using more challenging plasma samples. We found that the carbonate-bicarbonate solution (0.1 M, pH 11.3) was capable of releasing particles at LDL position without significant desorption of non-LDL fractions (Supplementary Fig. S5a), while the remaining non-LDL fractions were subsequently desorbed with NH_4_OH (0.28 M, pH 11.5) (Supplementary Fig. S5b). Thus, the carbonate-bicarbonate solution (0.1 M, pH 11.3) was selected to release LDL particles from the anti-apoB-100 disk, while NH_4_OH (0.28 M, pH 11.5) was chosen to desorb other chylomicrons and lipoproteins from the C6S disk. The NH_4_OH (0.28 M, pH 11.5) was also used to clean both disks.

#### Effect of active imidazole groups on non-specific adsorption of plasma proteins

Non-specific adsorption of undesirable particles onto stationary supports is a common issue in affinity chromatography. To minimize the non-specific interactions, the remaining active groups of the stationary support surface are usually deactivated. In this study, after selecting suitable eluents for the isolation system, we studied the effect of the deactivation of active imidazole groups on non-specific adsorption of plasma proteins. Firstly, the study was conducted with a blank CDI monolithic disk without any immobilized ligand. Without deactivation, the CDI disk non-specifically captured particles of different sizes with similar elution profiles comparable to those of major lipoproteins (Supplementary Fig. S6a). With deactivation with ethanolamine solution (2 M, pH 9), the overall number of the captured particles was greatly reduced (Supplementary Fig. S6b), proving that the deactivation was needed. The same deactivation studies were carried out after the attachment of the anti-apoB-100 mAb to the CDI disk. In this case, the deactivation also reduced non-specific adsorption of VLDL and other large protein aggregates towards the anti-apoB-100 disk as shown in Supplementary Fig. S7, demonstrating that the deactivation of the remaining active imidazole groups after the ligand immobilization was a crucial step to avoid non-specific adsorption, especially with complex biological fluids, such as plasma.

#### Determination of dynamic binding capacity of the anti-apoB-100 disk

To further investigate the ability of the anti-apoB-100 disk to capture LDL particles, we determined its dynamic binding capacity. Breakthrough curves were generated based on injections of ultracentrifugally isolated LDL with varying concentrations. To also further explore the effect of the deactivation, the breakthrough curves both before and after the deactivation of the anti-apoB-100 disk were plotted (Supplementary Figs S8a and S9a). The calculated dynamic binding capacity based on the amount of bound LDL per mL of monolithic disk at 50% breakthrough before deactivation was 1.50 mg/mL (Supplementary Fig. S8b). The estimated ligand utilisation was 0.062 µmol/µmol (Supplementary Table S3). After the deactivation of the anti-apoB-100 disk, we found that the dynamic binding capacity and the ligand utilisation of the disk were slightly increased to 1.66 mg/mL and 0.068 µmol/µmol, respectively (Supplementary Fig. S9). The dynamic binding curves with void volume were determined by the breakthrough of bovine serum albumin (BSA), which does not interact with the antibody. After correcting for void volume and normalizing the UV absorbance to the concentration, we found that the binding was independent of the LDL concentration in our selected concentration range (Supplementary Fig. S10).

#### Determination of suitable plasma volume and the isolation flow rate

Next, we determined the amount of plasma volume and the isolation flow rate to provide a relevant compromise between sample capacity and development of unfavorable backpressure. Based on the cholesterol analysis, loading volume of 220–420 µL of plasma seemed to be relevant, and thereby a loading of 320 µL of plasma diluted to 5 mL with PBS gave the optimal amount of bound cholesterol for analysis of the LDL fractions without the system backpressure (Supplementary Fig. S11). Furthermore, different flow rates ranging from 0.25 to 1.5 mL/min applied to the system resulted in no differences in the LDL-cholesterol (Supplementary Fig. S12), proving that the monolithic supports provide flow-unaffected binding capacity and efficiency as earlier reported^38–40^. Eventually, we selected 0.5 mL/min for the isolation flow rate to avoid backpressure that occurred during the experiments with multiple disks in a single housing. We also noted that non-fasting plasma samples might cause backpressure due to high concentrations of triglycerides and/or cholesterol, and the presence of concentrated chylomicrons.

### Affinity chromatographic isolation of LDL by C6S and anti-apoB-100 disks

Initial isolation of LDL was performed with a single anti-apoB-100 disk, and ELISA was exploited to quantify the level of apoB-100 and apoE present in LDL and other lipoproteins, such as chylomicrons, VLDL, IDL, and HDL particles. The results showed that the isolate from the anti-apoB-100 disk contained mainly apoB-100 with recovery of 91% and a small amounts of apoE (Supplementary Table S4). ApoE in the elutions suggested the co-binding of chylomicrons, VLDL particles, and VLDL remnants from plasma adsorbed to the anti-apoB-100 disk. Therefore, the second disk immobilized with C6S was needed to eliminate non-LDL particles from plasma samples. The final isolation system is shown in Fig. 1.

To isolate LDL with two disk system from plasma samples including relatively high level of chylomicrons and VLDL, separate housings can be employed to avoid system backpressure. Our two-disk system was also tested with the plasma having elevated chylomicrons and VLDL particles. The FPLC chromatograms of isolates from C6S and anti-apoB-100 disks can be found in Supplementary Fig. S13a,b. In addition, the cholesterol and the apoB-100 levels in the isolates from both C6S and anti-apoB-100 disk were measured. As shown in Fig. 2c, the fractions eluted at the apparent LDL position contained relatively high level of both cholesterol and apoB-100. ApoB-100 ELISA demonstrated 26% apoB-100 recovery for the anti-apoB-100 bound LDL, indicating successful and selective LDL isolation. The total protein recovery of both disks was 96% (572 µg) (Supplementary Table S5) when compared to the plasma apoB-100 content (598 µg). Thus, most of the apoB-100 containing lipoproteins (i.e. VLDL, IDL, LDL, and Lp(a)) were recovered by our system. The isolate from the C6S disk contained a substantial amount of proteins (420 µg) of different sizes (Supplementary Fig. S13a,c). The C6S disk clearly captured particles in the position of chylomicrons, VLDL, VLDL remnants including IDL, and these particles also contained a relatively small amount of cholesterol (Fig. 2b). While particles at LDL elution position were also present based on UV measurements (Supplementary Fig. S13a), these fractions contained very small amount of cholesterol and apoB-100 (Fig. 2b), and relatively high total protein level as compared to that of the isolate from anti-apoB-100 disk (Supplementary Fig. S13c). The findings suggest that the C6S may retain proteins and protein complexes that differed from LDL particles or contained Lp(a), a specific LDL type of particle attached with apo(a). To further examine the C6S-bound particles, we used the anti-apoB-100 antibody that differed from that of ELISA, i.e., anti-apoB-100 mAb attached to the anti-apoB-100 disk. After passing the C6S-bound particles through the anti-apoB100 disk, we found that the anti-apoB-100 disk only recovered 42% of the C6S-bound particles based on the UV-vis measurements at 280 nm (Supplementary Fig. S14). This supports the finding from ELISA result that the C6S-bound particles may also contain non-functional apoB-100, as they were unrecognised by apoB-100 ELISA.

**Figure 3:**
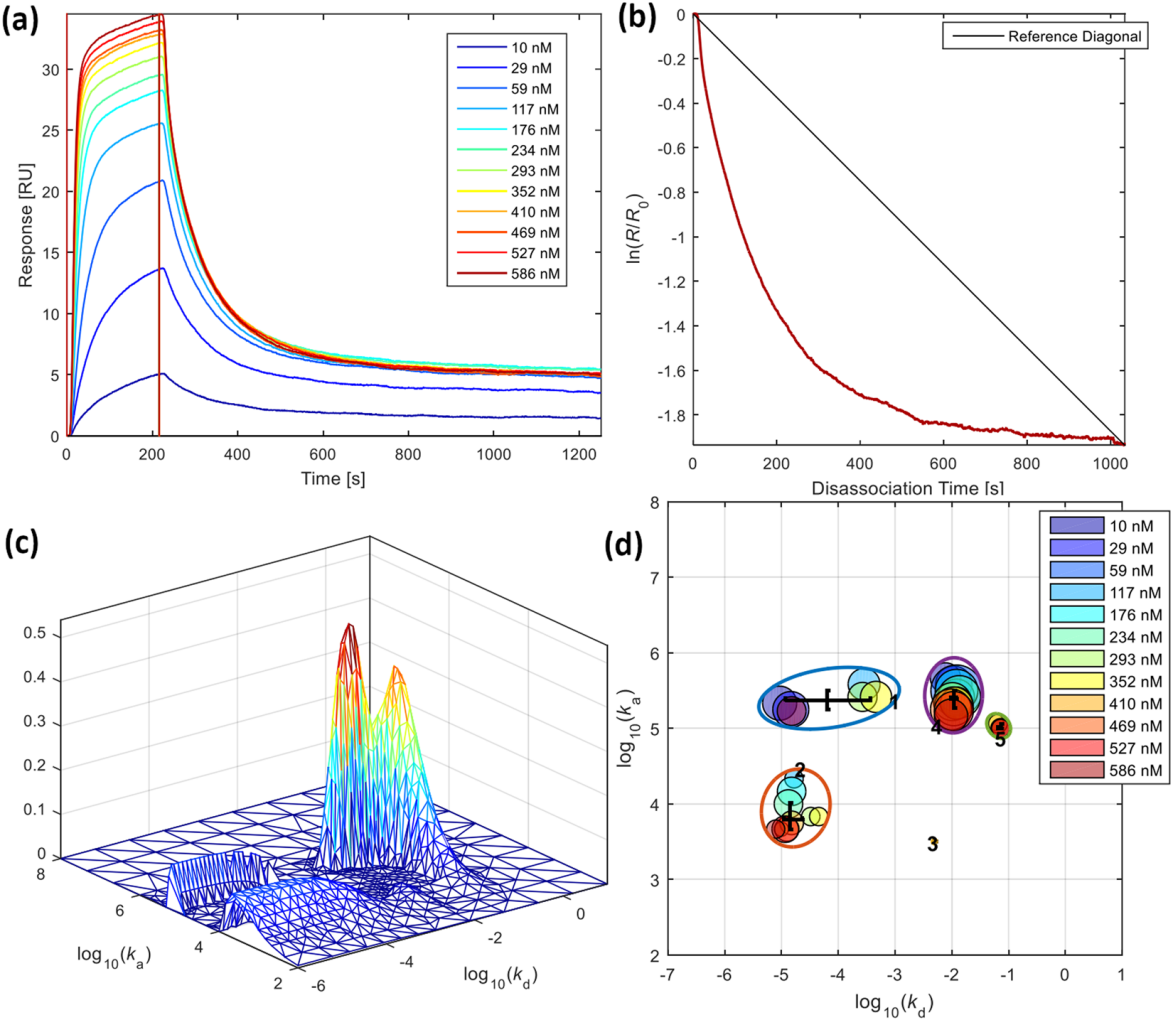
QCM studies of LDL isolated using the developed monolithic disk system (affinity chromatography): (**a**) representative adjusted sensorgrams for interactions between anti-apoB-100 mAb with LDL isolated using affinity chromatography method at different concentrations (nM); vertical line indicates injection end, (**b**) corresponding dissociation graph for a 586 nM LDL injection, (**c**) Rate Constants Distribution (RCD) for 586 nM, and (**d**) clustered rate constants. The unit nM indicates molar concentrations of LDL calculated using molecular weight of apoB-100 (512 kDa).

**Figure 4:**
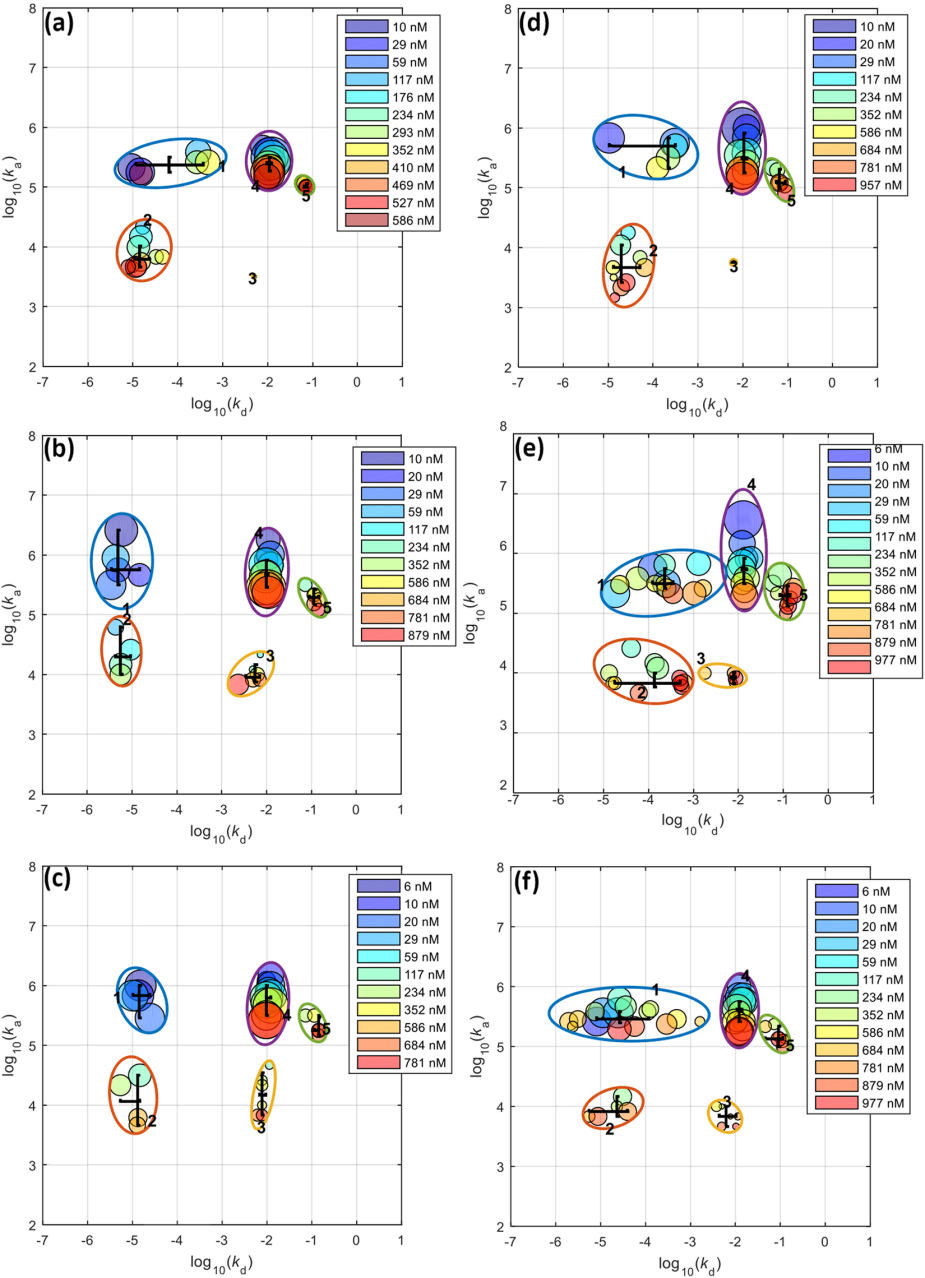
Clustered rate constants indicating five major interactions between anti-apoB-100 mAb and LDL isolated from two individuals using different isolation techniques: (**a**) individual #1 using monolithic disk system (affinity chromatography), (**b**,**c**) individual #1 using ultracentrifugation, (**d**) individual #2 using monolithic disk system (affinity chromatography), and (**e**,**f**) individual #2 using ultracentrifugation. The unit nM indicates molar concentrations of LDL calculated using molecular weight of apoB-100 (512 kDa).

**Figure 5:**
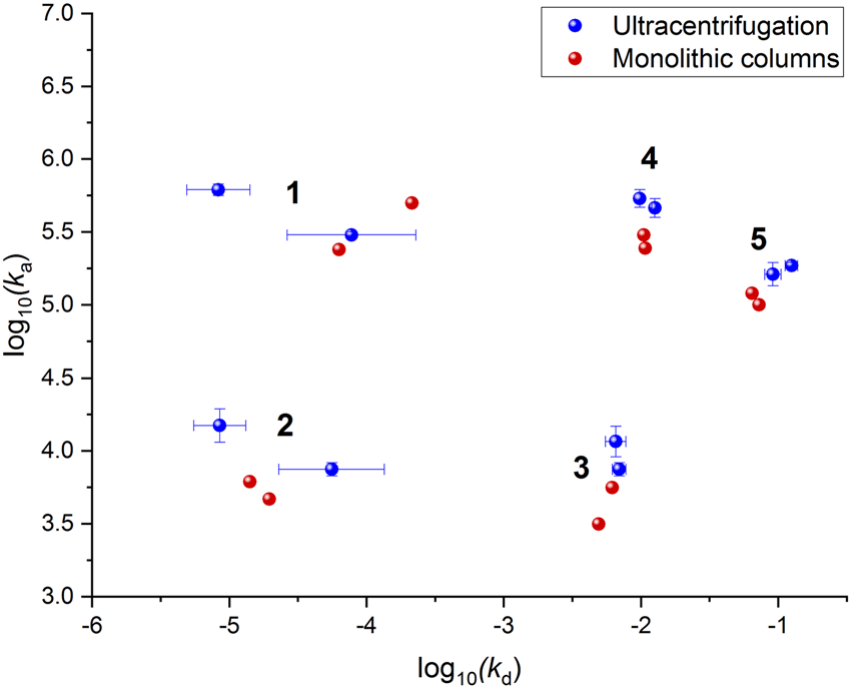
Clustered rate constants depicting five major interactions between anti-apoB-100 mAb and LDL of two individuals isolated using ultracentrifugation (blue) and monolithic disk system (affinity chromatography) (red), showing the similar rate constants regardless of the isolation methods used. Different dots represent different rate constants from each individual. Note that the rate constants of the interactions with anti-apoB-100 mAb with LDL isolated using ultracentrifugation were averaged. The association and dissociation constants can be found in Supplementary Table S6.

In addition to particle studies using FPLC, cholesterol, and ELISA analyses, we utilised quartz crystal microbalance (QCM), a reliable and sensitive method to investigate biomolecular interactions. The QCM was employed to assess LDL isolated from plasma of two healthy normolipidemic individuals with the two-disk system, compared to LDL obtained from the conventional ultracentrifugation. We adopted our previously used QCM protocol^37^ with a slight modification concerning the sample injection volume, resulting in longer association phase and more information to be achieved from the binding studies. The typical sensorgrams of the interaction between LDL and anti-apoB-100 mAb are shown in Supplementary Fig. S15 and Fig. 3a. As expected, the increase in response corresponded to the increase in LDL concentrations. Data analysis was performed using the Adaptive Interaction Distribution Algorithm (AIDA) developed by Forssén *et al*.^29^. The dissociation graph (Fig. 3b) indicated a heterogeneity in rate constants due to its deviation from the reference diagonal. The rest of the sensorgrams and dissociation curves of the experiments can be found in Supplementary Figs S16 and S17. The rate constant distributions (Fig. 3c) of LDL and the anti-apoB-100 mAb were successfully determined utilising AIDA and plotted as clusters shown in Fig. 3d. After comparing the rate constant distributions of the LDL samples isolated from our affinity system and ultracentrifugation (Fig. 4), we found a total of five clusters of rate constants (Supplementary Table S6) for all the LDL samples studied, representing five interactions in the system. The overlaid rate constants of these interactions for the isolated LDL are displayed in Fig. 5. It can be seen that the rate constants agree very well with each other, confirming that our newly developed affinity chromatographic method is selective for LDL isolation.

## Conclusions

We have successfully developed a selective isolation method for LDL particles from human plasma using two subsequent monolithic disks. The first C6S disk pre-isolated chylomicrons, VLDL, and VLDL remnants before the second anti-apoB-100 disk captured selectively LDL particles. In addition, the monolithic disk columns were reusable for subsequent isolations after the elution and appropriate washing steps. Some of the anti-apoB-100 disks were usable for over two years. Characteristics of the isolated LDL particles were similar to LDL from conventional ultracentrifugation, proving that the newly developed method enabled the selective isolation of pure LDL particles from small volume of plasma samples much faster compared to conventional ultracentrifugation and required less operator involvement. Potential automation of the method and transferring it to a microtiter plate format would increase the throughput and facilitate, e.g., functional studies of the isolated LDL. This aspect is of utmost importance since not only the level of plasma LDL cholesterol but functional properties of LDL particles are offering added value for evaluation of risks for coronary heart diseases^41^. Therefore, the LDL isolation method developed would offer the opportunity for new clinical applications and for diagnostic purposes.

## Methods

### Chemical and materials

Chemicals and materials used in the studies are listed in Supplementary section 3.1.

### Instrumentation

Instruments used in the studies can be found in Supplementary section 3.2.

### Preparation of solutions

Solutions were prepared as described in Supplementary section 3.3.

### Preparation of biological samples

Biological samples involved in the studies and their preparation can be found in Supplementary section 3.4. All experimental protocols were approved by the Ethical Committee of the University of Helsinki and performed in accordance with guidelines and regulations of the Finnish Advisory Board on Research Integrity (TENK). In addition, all plasma samples for lipoprotein isolation were provided by the Finnish Red Cross Blood Service with permission (Permission no. 37/2015). Informed consent was obtained from all participants (individual #1 and individual #2) for the QCM studies.

### Immobilization of affinity ligands onto monolithic disks

A commercial Convective Interaction Media® (CIM®) carbonyldiimidazole (CDI) monolithic disk (BIA Separations, Slovenia) (0.34 mL, pore size 1.3 µm, diameter 12 mm, thickness 3.0 mm) was integrated into a housing cartridge (BIA Separations, Slovenia). Prior to the immobilization, MilliQ water (6.8 mL) and PBS (15 mL, pH 7.4) were pumped through the disk by a syringe pump (SP100i, World Precision Instruments Inc., USA) to replace a storage solution (ethanol) and to equilibrate the disk, respectively. Throughout the immobilization process, the syringe pump was used at a flow rate of 0.5 mL/min, and the immobilization was performed at room temperature. In this study, two affinity ligands, anti-apoB-100 mAb (code Anti-h ApoB 2101 SPTN-5), which selectively recognizes human apoB-100 species, provided by Medix Biochemica Co. Inc. (Helsinki, Finland), and C6S (Sigma-Aldrich, USA), were immobilized onto two separate monolithic disks, namely anti-apoB-100 disk and C6S disk, respectively. To prepare the anti-apoB-100 disk, the anti-apoB-100 mAb solution (0.5 mg/mL, 5 mL) was pumped and recirculated through the disk for 3 h. The disk was allowed to stand in the housing for 20 h before washing with PBS (3 mL, pH 7.4) to remove unbound antibodies. Similarly, the C6S disk was prepared by recirculating C6S (5 mg/mL, 5 mL) through the disk for 3 h after equilibration. The disk was left to stand in the housing for 68 h before washing with PBS (3 mL, pH 7.4) to remove the unbound C6S. The remaining free imidazole groups on both disks were later deactivated by a manual injection of 2 M ethanolamine solution (2 mL, pH 9.0). The disks were incubated for 24 h and washed with PBS (3 mL, pH 7.4) to remove the remaining ethanolamine solution.

### Development of the isolation method

Experiments done during the isolation method development, including suitable affinity ligand selection, studies of CDI disk and non-specific adsorption, and determination of dynamic binding capacity, suitable elution solutions, flow rates, and suitable sample injection volumes, are described in Supplementary section 3.5.

### Fast protein liquid chromatography (FPLC) analysis

Plasma, lipoprotein samples, and isolated lipoprotein particles from C6S and anti-apoB-100 disks were fractionated using a Merck Hitachi FPLC system equipped with a pump model L-6200A (Merck, Darmstadt, Germany-Hitachi, Tokyo, Japan), a UV detector model L-4200, an integrator model D-7500, and a manual injector with 1.0 mL sample loop. Analyses of the lipoprotein particles were carried out on a Superose® 6 HR 10/300 size-exclusion chromatography column with PBS (pH 7.4) as mobile phase at a flow rate of 0.5 mL/min. The detection wavelength was set at 280 nm to preliminarily measure proteins present in the samples. Samples were manually injected using a Hamilton syringe. Total run time for of the experiments was 40 min. Fractions (0.5 mL each) for further analyses were collected using a fraction collector (Retriever® 500).

### Isolation of low-density lipoprotein (LDL) using affinity monolithic disks

Prior to the actual LDL isolation using both C6S and anti-apoB-100 disk system, preliminary studies were performed with a single anti-apoB-100 disk. An undiluted non-fasting plasma sample (1 mL) was manually injected with a 1-mL syringe to the anti-apoB-100 disk placed in a housing, followed by PBS wash (10 mL) and elution with 4 x NH_4_OH (1 mL, 0.28 M, pH 11.5). The levels of apoB-100 and apoE in each isolate and plasma sample were measured. Thereafter, the isolation of LDL from human blood plasma was carried out using two monolithic disks arranged in tandem with the C6S disk being the first disk to contact plasma, followed by the anti-apoB-100 disk. To isolate LDL from plasma samples with elevated levels of chylomicrons, VLDL, and VLDL remnants including IDL, the disks could also be placed in tandem in separate housings to avoid system backpressure. To prove the concept, we used plasma with elevated levels of the mentioned lipoproteins (320 µL). The plasma was diluted with PBS (4.68 mL, pH 7.4) and injected to the isolation system through a 5-mL syringe using the syringe pump. Flow-through (unbound) plasma was collected, followed by PBS wash (2 ml, pH 7.4). The isolation was done in replicate, and the isolates were combined. Desorption of bound analyte was performed using NH_4_OH (1.5 mL, 0.28 M, pH 11.5) for the C6S disk and carbonate-bicarbonate solution (1.5 mL, 0.1 M, pH 11.3) for the anti-apoB-100 disk. The isolates obtained were neutralized right after desorption with HCl. After the desorption of bound particles with carbonate-bicarbonate solution, the anti-apoB-100 disk was washed with PBS (2 mL, pH 7.4) and NH_4_OH (1.5 mL, 0.28 M, pH 11.5) to remove remaining bound particles. Both disks were equilibrated with PBS (2 mL, pH 7.4). Additional binding experiments are described in Supplementary section 3.6.

### Total cholesterol analysis

Total cholesterol (free and esterified cholesterol) levels in the samples were measured using Roche Cholesterol CHOD-PAP reagent (kit no. 1489232; Roche, Germany) according to manufacturer’s instruction. The absorbance was measured at 510 nm EnSpire® Multimode Plate Reader (PerkinElmer Inc., USA). Calibration curves and sample concentrations were analysed using EnSpire multilabel analyser version 4.13.3005.1482. The amount of cholesterol (mg) was calculated from the cholesterol molar mass (387 g/mol)^42^.

### Total protein analysis

Protein levels in the samples were measured using Bio-Rad DC™ Protein Assay kit (Bio-Rad Laboratories, Hercules, CA). Measurements were performed according to the assay protocol. The protein concentration was measured at 750 nm using the plate reader as described in the above section.

### Apolipoprotein B-100 and apolipoprotein E enzyme-linked immunosorbent assays (ELISAs)

Detection and quantification of apoB-100 and apoE in the samples was carried out using a human apolipoprotein B ELISA^PRO^ kit (Code 3715-1HP-2, Mabtech AB, Sweden) and apoE ELISA kits (Code 3712-1H-6, Mabtech AB, Sweden) according to manufacturer’s protocols. The measurements were done at 450 nm. For apoB-100 ELISA, only fractions eluting at 13 to 26 min from the FPLC were analysed.

### Quartz crystal microbalance studies

The immobilization of anti-apoB-100 mAb onto an LNB-carboxyl sensor chip was performed with the amine coupling procedure as described in^37^ with slight modifications of the injection volumes. Prior to the immobilization, the LNB-carboxyl sensor chip was pre-wetted *ex situ* with 20 μL of MilliQ water using a pipette. The pre-wetted sensor chip was inserted to the instrument and left to stabilize in HEPES buffer (*I* = 10 mM, 150 mM NaCl, 0.005% TWEEN 20, pH 7.4) until a stable baseline was achieved. All of the subsequent immobilization steps were performed at 10 μL/min under 25 °C. Surface activation was done by injecting 100 μL of a freshly prepared mixture of 0.2 M EDC and 0.05 M S-NHS solutions (1:1, v/v) for 300 s. All injections were achieved using an automated C-Fast software. Immobilization of anti-apoB-100 mAb (0.1 mg/mL) was carried out by two consecutive injections of anti-apoB-100 solution (100 µL, 100 μg/mL, in PBS, pH 6.4) for 300 s each. After the coupling, the remaining active carboxyl groups were deactivated with two injections of 1 M ethanolamine solution (pH 9.0) for 300 s each. The kinetic measurements with the QCM biosensor utilised LDL samples isolated from plasma of two healthy individuals using conventional ultracentrifugation protocols and our affinity-based approach. All experiments were done at 25 μL/min under 37 °C with the sample injection volume of 90 μL. The LDL samples (concentration range: 0–500 µg/mL) were injected to an LNB-carboxyl sensor chip immobilized with anti-apoB-100 mAb using C-Fast software under running buffer (PBS). The injected samples were allowed to dissociate for 1000 s. Thereafter, regeneration of the sensor chip surface was done by the injection of 90 μL of 0.28 M NH_4_OH solution (pH 11.5). This was followed by injection of 90 µL of PBS (pH 7.4) before the next sample injection. Sensorgrams recorded were visualised using Attana Evaluation software.

### Biosensor data analysis

The biosensor data sets were analysed with respect to binding site heterogeneity using the numerical algorithm, namely Adaptive Interaction Distribution Algorithm (AIDA), as described in detail in^29^. In brief, the data analysis involves the following steps. Firstly, the binding site heterogeneity was tested by inspecting the dissociation plot to determine the interaction model. Thereafter, the rate constant distribution is calculated by fitting each of the experimentally obtained sensorgram individually. Finally, the emerging rate constants are plotted and subjected to a cluster analysis, giving major clusters of all the rate constants^29^. The unit concentrations of VLDL, IDL, and LDL (nM) were calculated using molecular weight of apoB-100 (512 kDa).

## Supplementary information

Rapid affinity chromatographic isolation method for LDL in human plasma by immobilized chondroitin-6-sulfate and anti-apoB-100 antibody monolithic disks in tandem

## Data Availability

The datasets generated and analysed during the current study are available from the corresponding author on reasonable request.
