# Supplementary material for: Rapid affinity chromatographic isolation method for LDL in human plasma by immobilized chondroitin-6-sulfate and anti-apoB-100 antibody monolithic disks in tandem
Source: Sci Rep. 2019 Aug 2;9:11235. doi: 10.1038/s41598-019-47750-z (PMC6677805; doi:10.1038/s41598-019-47750-z)
Supplement: Supplementary file 1 — Rapid affinity chromatographic isolation method for LDL in human plasma by immobilized chondroitin-6-sulfate and anti-apoB-100 antibody monolithic disks in tandem [file 41598_2019_47750_MOESM1_ESM.pdf]

Supplementary Information

Rapid affinity chromatographic isolation method for LDL in human plasma by immobilized chondroitin-6-sulfate and anti-apoB-100 antibody monolithic disks in tandem

Thanaporn Liangsupree[1], Evgen Multia[1], Jari Metso[2], Matti Jauhiainen[2], Patrik Forssén[3], Torgny Fornstedt[3], Katariina Öörni[4], Aleš Podgornik[5,6], Marja-Liisa Riekkola[1*]

[1]Department of Chemistry, P.O. Box 55, FI-00014 University of Helsinki, Finland.

[2]Minerva Foundation Institute for Medical Research and National Institute for Health and Welfare, Biomedicum 2U, Helsinki, Finland.

[3]Department of Engineering and Chemical Sciences, Karlstad University, SE-651 88 Karlstad, Sweden.

[4]Wihuri Research Institute, Atherosclerosis Research Laboratory, Haartmaninkatu 8, 00290 Helsinki, Finland.

[5]Faculty of Chemistry and Chemical Technology, University of Ljubljana, Ljubljana, Slovenia.

[6]Center of Excellence for Biosensors, Instrumentation and Process Control - COBIK, Ajdovščina, Slovenia.

*Correspondence and requests for materials should be addressed to M.-L. R. (E-mail: marja-liisa.riekkola@helsinki.fi)

Low-density lipoprotein (LDL) is considered the major risk factor for the development of atherosclerotic cardiovascular diseases (ASCVDs). A novel and rapid method for the isolation of LDL from human plasma was developed utilising affinity chromatography with monolithic stationary supports. The isolation method consisted of two polymeric monolithic disk columns, one immobilized with chondroitin-6-sulfate (C6S) and the other with apolipoprotein B-100 monoclonal antibody (anti-apoB-100 mAb). The first disk with C6S was targeted to remove chylomicrons, very-low-density lipoprotein (VLDL) particles, and their remnants including intermediate-density lipoprotein (IDL) particles, thus allowing the remaining major lipoprotein species, i.e. LDL, lipoprotein(a) (Lp(a)), and high-density lipoprotein (HDL) to flow to the anti-apoB-100 disk. The second disk captured LDL particles via the anti-apoB-100 mAb attached on the disk surface in a highly specific manner, permitting the selective LDL isolation. The success of LDL isolation was confirmed by different techniques including quartz crystal microbalance. In addition, the method developed gave comparable results with ultracentrifugation, conventionally used as a standard method. The reliable results achieved together with a short isolation time (less than 30 min) suggest the method to be suitable for clinically relevant LDL functional assays.

Supplementary section 1: Development of the isolation method

1.1 QCM studies of the interactions between VLDL-IDL mixture and C6S

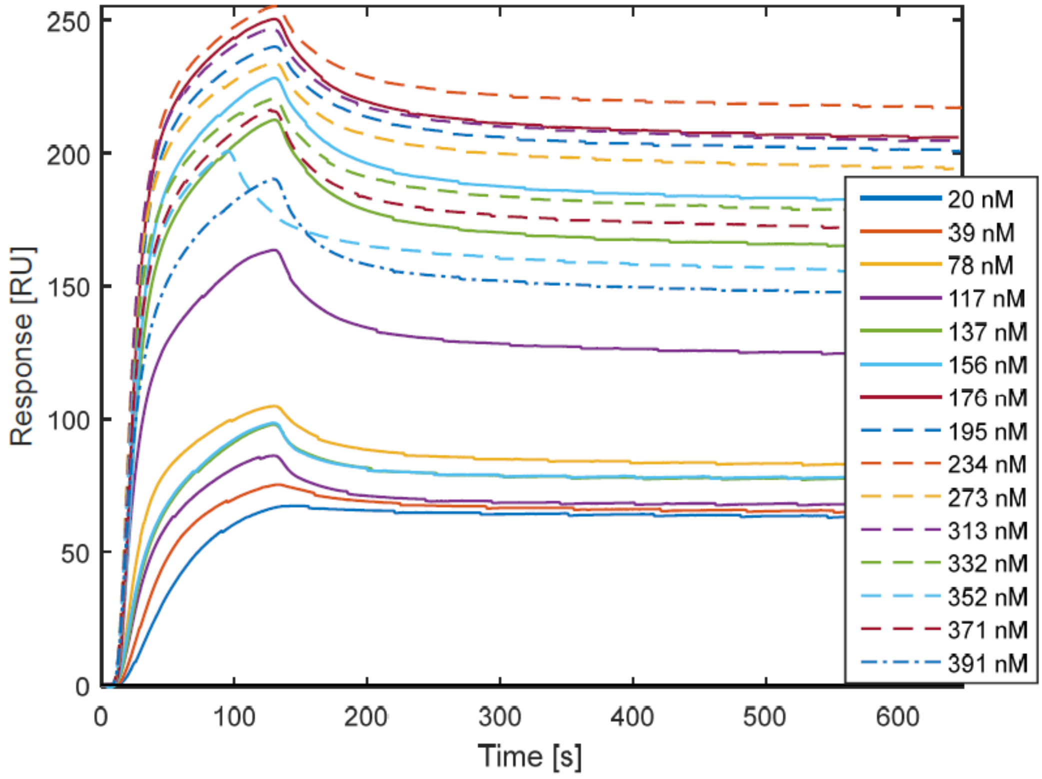

Figure S1. Adjusted sensorgrams for the interactions between different concentrations of ultracentrifugally purified VLDL-IDL mixture and C6S ligand immobilized on the LNB-carboxyl sensor chip. The VLDL-IDL mixture contained chylomicrons, VLDL, and VLDL remnants, including IDL particles. The unit nM indicates molar concentrations of VLDL and IDL calculated using molecular weight of apoB-100 (512 kDa).

1.2 FPLC chromatograms of plasma sample and ultracentrifugally isolated lipoprotein particles (LDL and VLDL-IDL mixture)

(a) Plasma

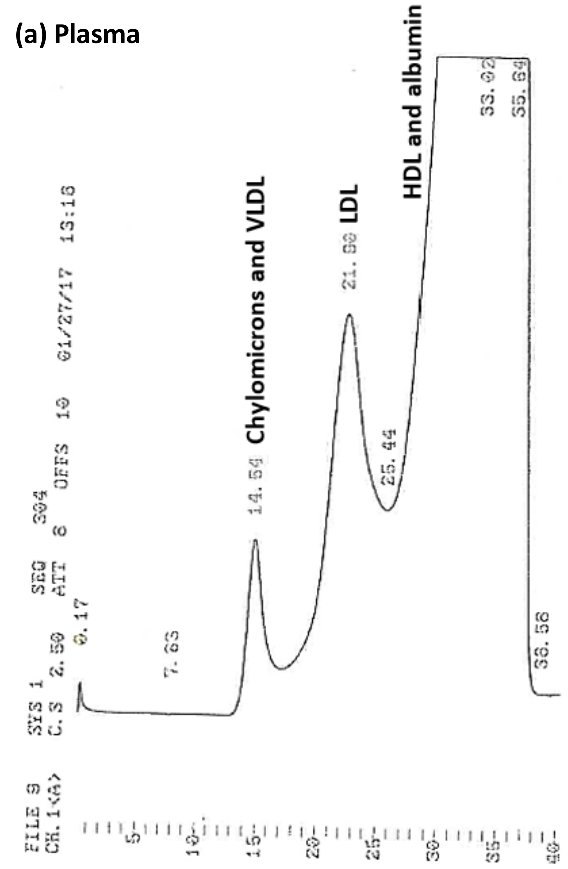

Peak areas (detection at 280 nm)

FILE : 8 (00/00/00 00:00)
CALC-METHOD: AR/HI% <AREA> COMPONENT

NO.	RT	AREA	CONC	BC
1	0.17	215962	0.082	BB
2	7.83	129	0.000	BB
3	8.12	166	0.000	BB
4	8.32	277	0.000	BB
5	14.54	6405069	2.425	BV
6	21.90	35066926	13.279	VV
7	25.44	406005	0.154	VV
8	33.02	136126529	51.546	VV
9	33.16	6117924	2.317	VV
10	33.20	75533319	28.601	VV
11	35.64	106572	0.040	TBV
12	35.84	115650	0.044	TVV
13	35.92	277304	0.105	TVV
14	36.03	251550	0.095	TVV
15	36.11	504610	0.191	TVV
16	36.22	2948959	1.117	TVB
17	36.56	255	0.000	TBB
18	38.86	9972	0.004	VB
TOTAL				
		264088560	100.000	

(b) VLDL-IDL

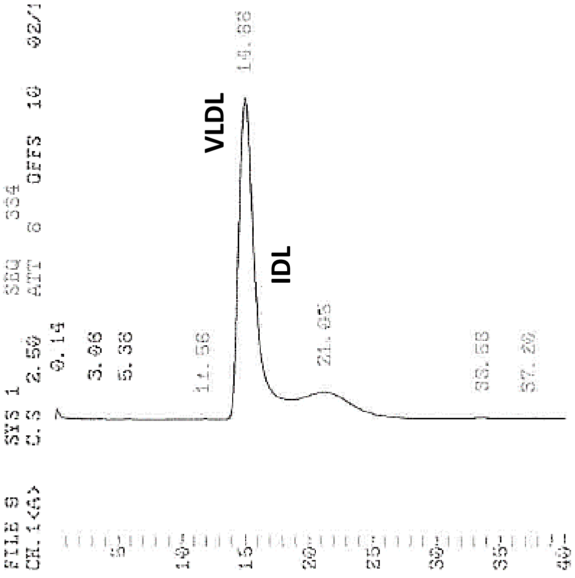

Peak areas (detection at 280 nm)

FILE : 8 (00/00/00 00:00)
CALC-METHOD: AR/HI% <AREA> COMPONENT TBL

NO.	RT	AREA	CONC	BC
1	0.14	50609	0.404	BB
2	3.06	512	0.004	BB
3	5.36	1254	0.010	BV
4	5.81	7281	0.058	VB
5	11.56	566	0.005	BB
6	12.49	642	0.005	BB
7	14.88	11452007	91.413	BV
8	21.05	939959	7.503	TBB
9	33.58	74656	0.596	TBB
10	37.20	162	0.001	TBB
TOTAL				
		12527630	100.000	

PEAK REJ : 0

Figure S2. FPLC chromatograms and peak areas of (a) plasma (320 µL injected), (b) VLDL-IDL mixture containing chylomicrons, VLDL, and VLDL remnants, including IDL particles, (320 µL injected), and (c) LDL (500 µL injected, 200 µg of LDL protein). Both lipoprotein standards were isolated from plasma samples using ultracentrifugation. FPLC flow rate was 0.5 mL/min. Detection was done at $A_{280\ nm}$.

(c) LDL

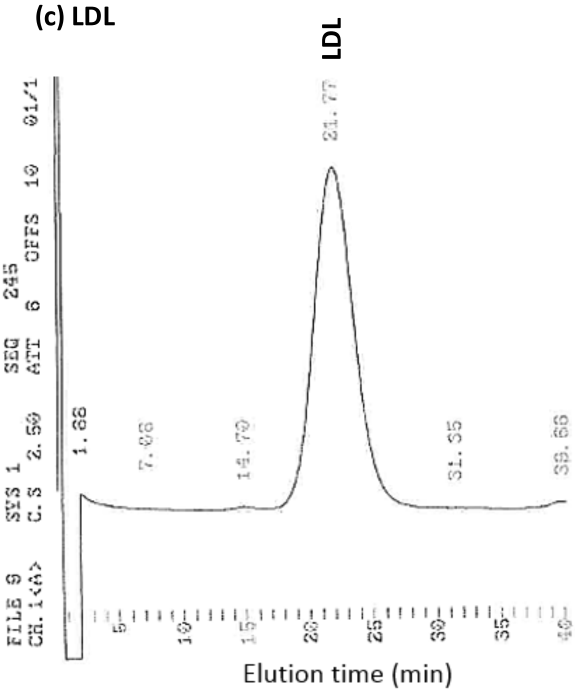

Peak areas (detection at 280 nm)

FILE : 9 (00/00/00 00:00)
CALC-METHOD: AR/HI% <AREA> COMPONENT

NO.	RT	AREA	CONC	BC
1	0.16	1329650	4.090	BB
2	1.68	6563640	20.193	BV
3	7.06	5746977	17.686	VV
4	14.70	3441413	10.587	VV
5	21.77	15302441	47.076	VV
6	31.35	2211	0.007	TBB
7	39.66	117465	0.361	VB
TOTAL		32505997	100.000	

Figure S2 (continued). FPLC chromatograms and peak areas of (a) plasma (320 µL injected), (b) VLDL-IDL mixture containing chylomicrons, VLDL, and VLDL remnants, including IDL particles, (320 µL injected), and (c) LDL (500 µL injected, 200 µg of LDL protein). Both lipoprotein standards were isolated from plasma samples using ultracentrifugation. FPLC flow rate was 0.5 mL/min. Detection was done at $A_{280\ nm}$.

1.3 Determination of suitable eluents for C6S and anti-apoB-100 disks

Table S1. Eluents and results for the determination of suitable eluents for the C6S disk.

Condition	Eluent	Success in desorbing bound proteins
**Ionic strength**	2 M NaCl	No
**High pH**	0.1 M carbonate-bicarbonate solution, pH 11.3	Partially
**High pH**	0.28 M NH_4OH, pH 11.5	Yes

Table S2. Eluents and results for the determination of suitable eluents for the anti-apoB-100 disk.

Condition	Eluent	Success in desorbing DiI[(a)]-LDL
**Ionic strength**	1 M KCl	No
**Ionic strength**	3 M KCl	No
**Low pH**	100 mM glycine-HCl, pH 3.0	No
**Low pH**	100 mM citric acid, pH 3.0	No
**High pH**	0.28 M NH_4OH, pH 11.2	No
**High pH**	0.28 M NH_4OH, pH 11.5	Yes

[(a)]1,1'-Dioctadecyl-3,3,3',3'-Tetramethylindocarbocyanine Perchlorate

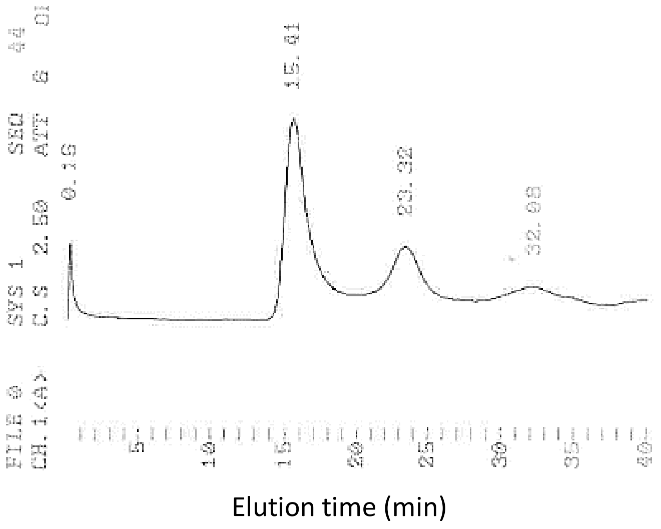

<u>Peak areas (detection at 280 nm)</u>

FILE : 0
CALC-METHOD: AR/HI% <AREA> COMPONENT

NO.	RT	AREA	CONC	BC
1	0.16	117543	3.468	BB
2	15.41	1749705	51.623	BV
3	23.32	1070750	31.591	VV
4	32.08	451372	13.317	VB
TOTAL		3389370	100.000	

PEAK REJ : 0

Figure S3. FPLC chromatogram and peak areas of the C6S-bound particles. The eluent was NH_4OH (0.28 M, pH 11.5). The VLDL-IDL mixture isolated from plasma using ultracentrifugation injected to the C6S disk was 200 µL of the mixture diluted to 5 mL with PBS (pH 7.4). The FPLC and isolation flow rates for the C6S disk were 0.5 mL/min. Detection was done at $A_{280\ nm}$.

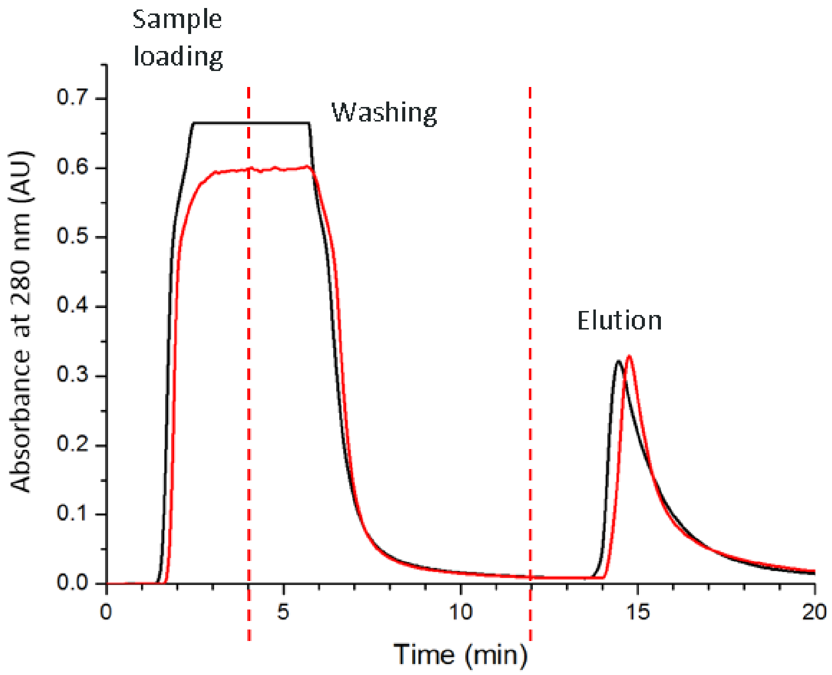

Figure S4. Desorption profiles of the anti-apoB-100 disk before (red) and after (black) deactivation of the active imidazole groups. The original plasma sample was 1 mL. The eluent was NH_4OH (0.28 M, pH 11.5). Detection was done at $A_{280\ nm}$.

1.4 The desorption of bound particles on the anti-apoB-100 disk

(a)

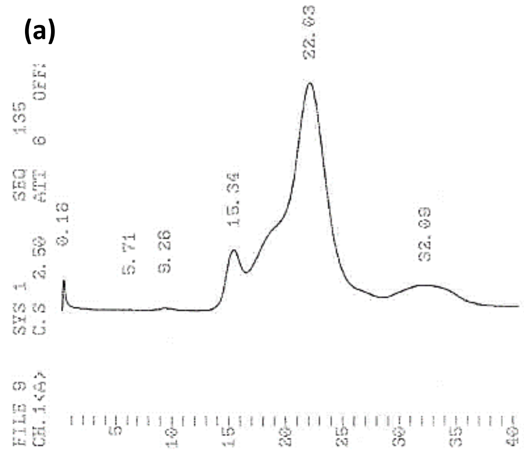

Elution time (min)

Peak areas (detection at 280 nm)

FILE : 9 (00/00/00 00:00)
CALC-METHOD: AR/HI% <AREA> COMPONENT

NO.	RT	AREA	CONC	BC
1	0.16	57374	0.828	BB
2	5.71	2173	0.031	BB
3	9.26	14753	0.212	BB
4	15.34	545226	7.851	BV
5	22.03	5904756	85.027	VV
6	32.09	420303	6.052	TBB
TOTAL		6944585	100.000	

PEAK REJ : 0

(b)

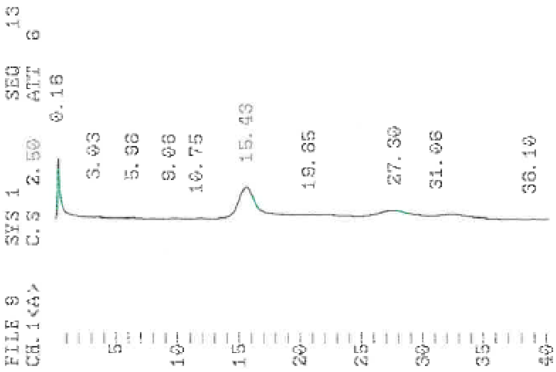

Elution time (min)

Peak areas (detection at 280 nm)

FILE : 9 (00/00/00 00:00)
CALC-METHOD: AR/HI% <AREA> COMPONENT

NO.	RT	AREA	CONC	BC
1	0.16	105354	23.231	BV
2	3.03	776	0.171	TBB
3	5.96	1031	0.227	BB
4	9.06	145	0.032	BB
5	10.75	74	0.016	BB
6	15.43	237692	52.456	BB
7	19.65	915	0.202	BV
8	20.64	5792	1.277	VB
9	27.30	71613	15.791	BB
10	31.06	489	0.108	BV
11	32.31	28506	6.286	VB
12	36.10	921	0.203	BB
TOTAL		453510	100.000	

PEAK REJ : 0

Figure S5. FPLC chromatograms and peak areas of (a) the anti-apoB-100-bound particles using carbonate-bicarbonate (0.1 M, pH 11.3) as an eluent and (b) fraction containing particles washed with NH_4OH (0.28 M, pH 11.5) after carbonate-bicarbonate wash. The plasma sample was 400 µL diluted to the final volume of 5 mL with PBS. The isolation flow rate for the anti-apoB-100 disk was 0.25 mL/min, and FPLC flow rate was 0.5 mL/min. Detection was done at $A_{280\ nm}$.

1.5 Effect of the deactivation of CDI groups on monolithic disks

(a) Isolate from CDI disk before deactivation

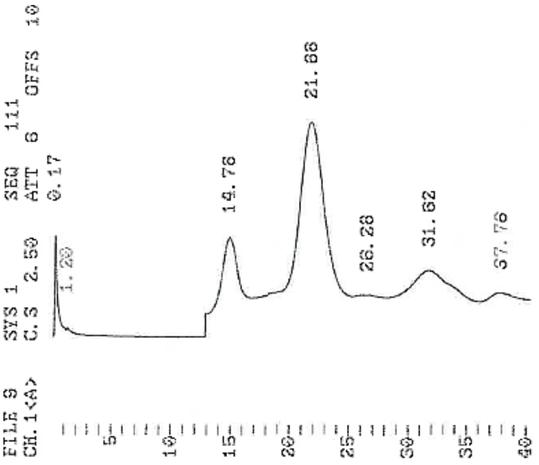

Peak areas (detection at 280 nm)

FILE : 9 (00/00/00 00:00)
CALC-METHOD: AR/HI% <AREA> COMPONENT

NO.	RT	AREA	CONC	BC
1	0.17	189542	3.073	BV
2	1.20	2532	0.041	TBB
3	14.78	1068863	17.328	BV
4	21.68	3758815	60.932	VV
5	26.28	17046	0.276	TBB
6	31.62	1003784	16.273	VV
7	37.78	128094	2.077	VB
TOTAL		6168499	100.000	

PEAK REJ : 0

(b) Isolate from CDI disk after deactivation

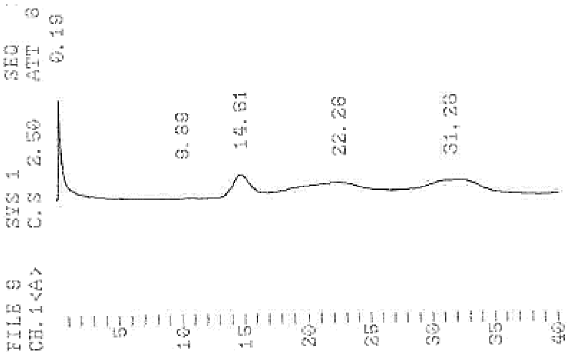

Peak areas (detection at 280 nm)

FILE : 9 (00/00/00 00:00)
CALC-METHOD: AR/HI% <AREA> COMPONENT TBL

NO.	RT	AREA	CONC	BC
1	0.19	199571	28.169	BB
2	9.69	576	0.081	BB
3	14.61	157368	22.231	BV
4	15.51	34072	4.813	VV
5	22.26	277720	39.228	VB
6	31.26	19776	2.793	BB
7	32.26	18880	2.664	BB
TOTAL		707983	100.000	

PEAK REJ : 0

Figure S6. FPLC chromatograms and peak areas of the eluates from the CDI disk before (a) and after (b) deactivation with ethanolamine (2 M, pH 9.0). The plasma sample was 600 µL diluted to the total volume of 1.2 mL with PBS. The eluent was NH_4OH (0.28 M, pH 11.5). The flow rate for the CDI disk was 0.25 mL/min. FPLC flow rate was 0.5 mL/min. Detection was done at $A_{280\ nm}$.

(a) Isolate from anti-apoB-100 disk before deactivation

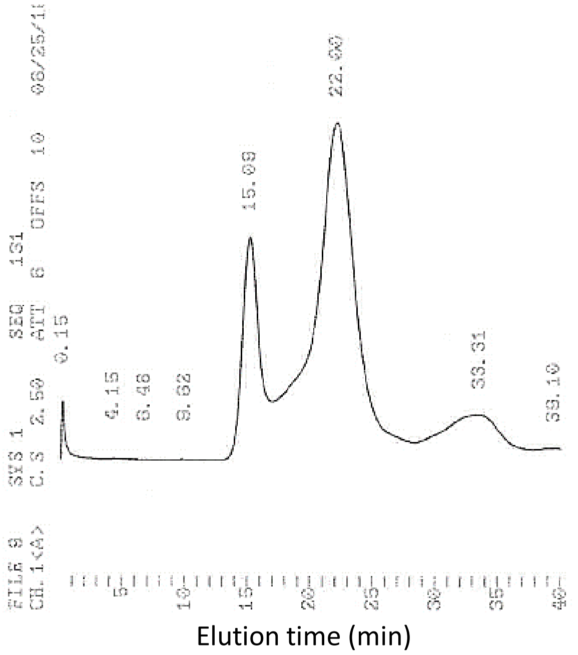

Peak areas (detection at 280 nm)

FILE : 9 (00/00/00 00:00)
CALC-METHOD: AR/HI% <AREA> COMPONENT TBL

NO.	RT	AREA	CONC	BC
1	0.15	98969	1.063	BB
2	4.15	2166	0.023	TBB
3	6.46	25	0.000	BB
4	9.62	963	0.010	BB
5	15.09	1800001	19.326	BV
6	22.00	6431818	69.057	VV
7	33.31	970549	10.421	VV
8	39.10	9215	0.099	VB
TOTAL		9313748	100.000	

PEAK REJ : 0

(b) Isolate from anti-apoB-100 disk after deactivation

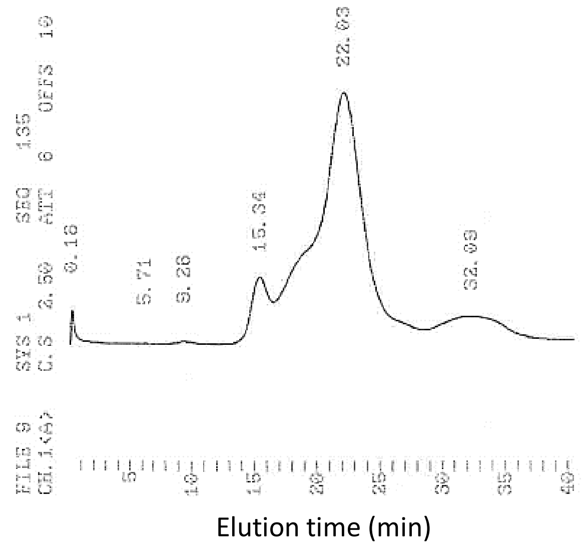

Peak areas (detection at 280 nm)

FILE : 9 (00/00/00 00:00)
CALC-METHOD: AR/HI% <AREA> COMPONENT

NO.	RT	AREA	CONC	BC
1	0.16	57374	0.826	BB
2	5.71	2173	0.031	BB
3	9.26	14753	0.212	BB
4	15.34	545226	7.851	BV
5	22.03	5904756	85.027	VV
6	32.09	420303	6.052	TBB
TOTAL		6944585	100.000	

PEAK REJ : 0

Figure S7. FPLC chromatograms and peak areas of fractionated lipoprotein particles from the anti-apoB-100 disk before (a) and after (b) deactivation. The plasma sample injected to the anti-apoB-100 disk was 400 µL diluted to the total volume of 5 mL with PBS. The eluent was carbonate-bicarbonate solution (0.1 M, pH 11.3). The flow rate for the anti-apoB-100 disk was 0.25 mL/min. FPLC flow rate was 0.5 mL/min. FPLC detection was done at $A_{280\ nm}$. (c) Cholesterol profiles (total cholesterol content in mg) of fractionated lipoprotein particles from the anti-apoB-100 disk before and after deactivation.

(c) Cholesterol profiles

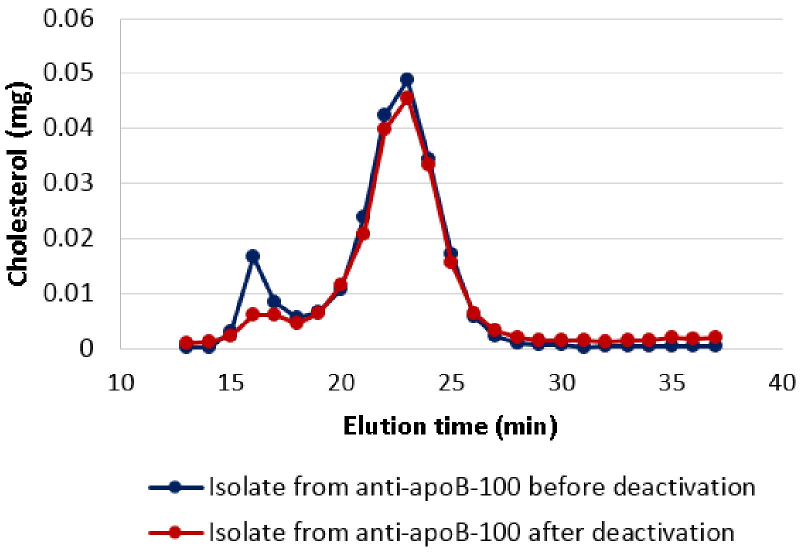

Figure S7 (continued). FPLC chromatograms and peak areas of fractionated lipoprotein particles from the anti-apoB-100 disk before (a) and after (b) deactivation. The plasma sample injected to the anti-apoB-100 disk was 400 µL diluted to the total volume of 5 mL with PBS. The eluent was carbonate-bicarbonate solution (0.1 M, pH 11.3). The flow rate for the anti-apoB-100 disk was 0.25 mL/min. FPLC flow rate was 0.5 mL/min. FPLC detection was done at $A_{280\ nm}$. (c) Cholesterol profiles (total cholesterol content in mg) of fractionated lipoprotein particles from the anti-apoB-100 disk before and after deactivation.

1.6 Dynamic binding capacity of the anti-apoB-100 disk

(a)

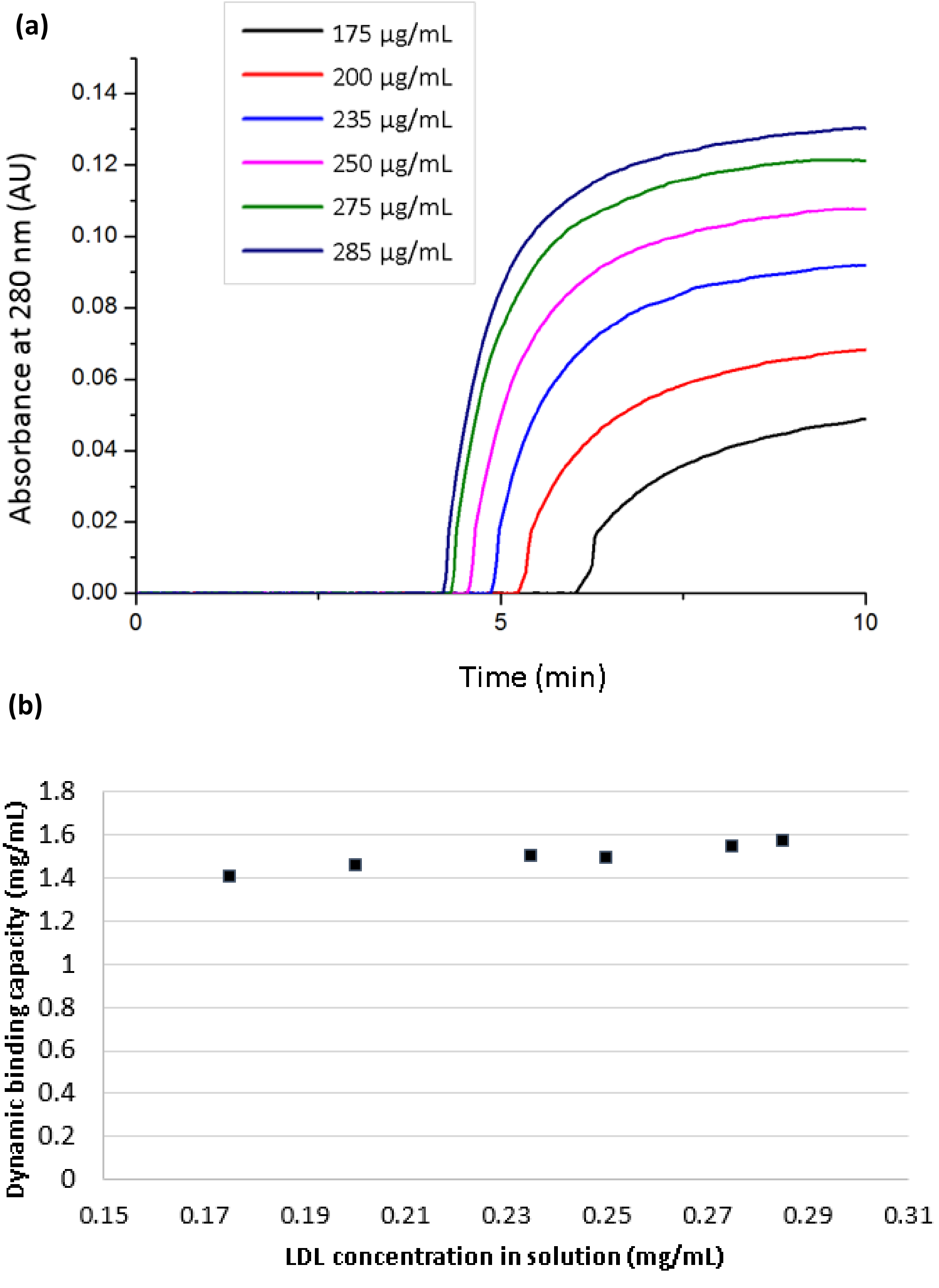

Figure S8. (a) Breakthrough curves obtained from injections of different concentrations of LDL ranging from 175 µg/mL to 285 µg/mL through the anti-apoB-100 disk before deactivation. The concentrations were based on the total protein content in LDL. (b) Dynamic binding capacity based on the amount of bound LDL per mL support at 50% breakthrough. Detection was done at $A_{280\ nm}$. The flow rate used was 0.5 mL/min.

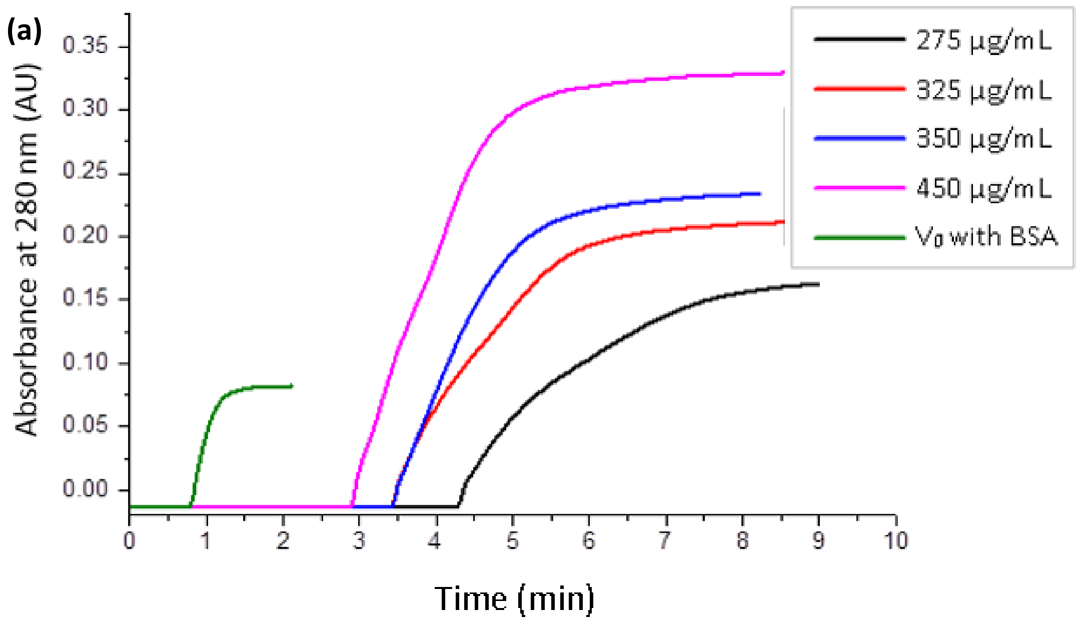

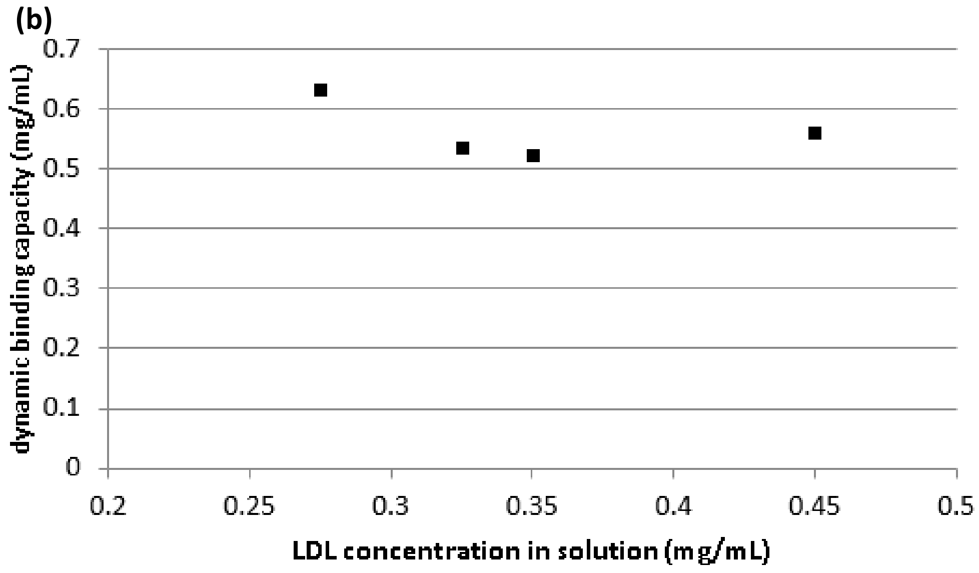

Figure S9. (a) Breakthrough curves obtained from injections of BSA and different concentrations of LDL ranging from 275 µg/mL to 450 µg/mL through the anti-apoB-100 disk after deactivation. The concentrations were based on the total protein content in LDL. (b) Dynamic binding capacity of the anti-apoB-100 disk after deactivation calculated based on the amount of bound LDL per mL support at 50% breakthrough. Detection was done at $A_{280\ nm}$. The flow rate used was 0.5 mL/min.

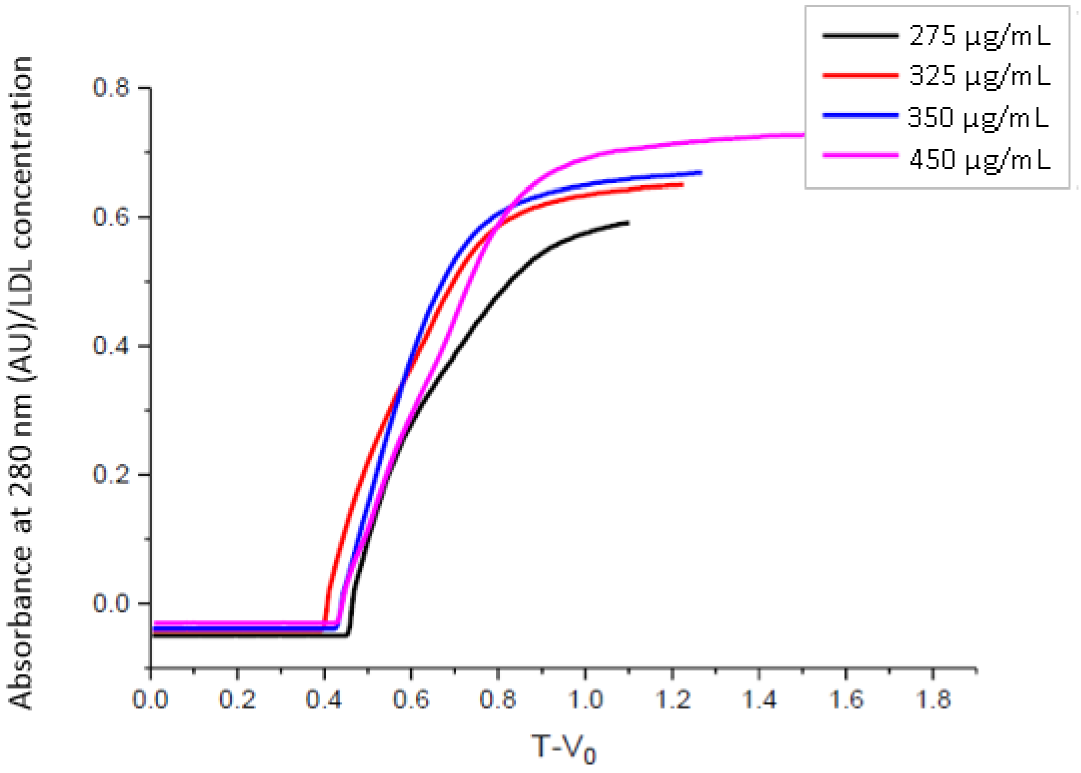

Figure S10. Breakthrough curves at different LDL concentrations of the anti-apoB-100 disk after deactivation adjusted for the void volume by BSA and concentrations. Detection was done at $A_{280\ nm}$. The flow rate used was 0.5 mL/min.

Table S3. Ligand density and dynamic binding capacity for the anti-apoB-100 disk based on the amount of bound LDL per mL support at 50% breakthrough before and after deactivation of the CDI groups.

**Anti-apoB-100 disk**	**Ligand density**		**Dynamic binding capacity**		**Ligand utilisation**
	**[mg/mL]**	**µmol[(a)]**	**[mg/mL]**	**µmol[(b)]**	**[µmol/µmol]**
Before deactivation	0.5	0.016	1.50	$9.97*10^{-4}$	0.062
After deactivation	0.5	0.016	1.66	$1.11*10^{-3}$	0.068

[(a)]Calculated based on 2.5 mg of anti-apoB-100 mAb injected to the disk. The molar mass of the anti-apoB-100 mAb is 155000 g/mol.

[(b)]Calculated based on the amount of bound LDL at 50% breakthrough per disk (0.34mL). The molar mass of apoB-100 is 512000 g/mol.

1.7 Determination of the suitable plasma volume for the isolation of LDL

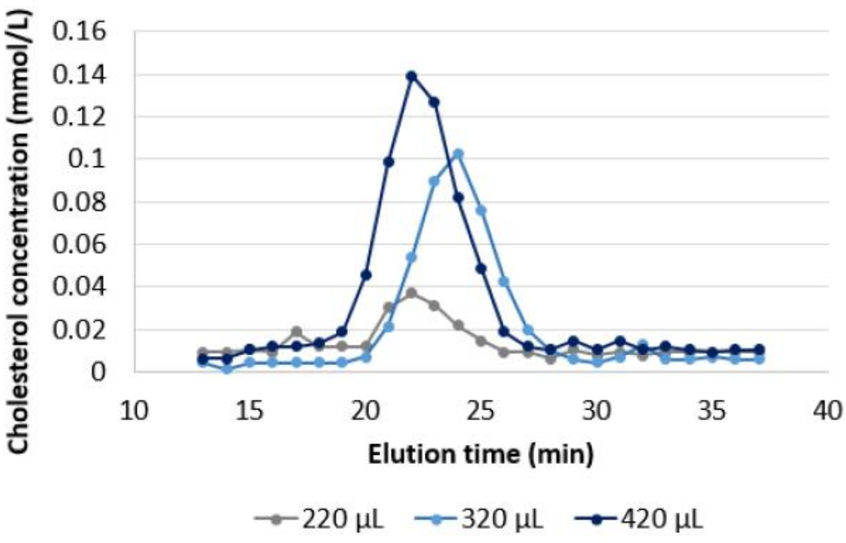

Figure S11. FPLC profiles of LDL particles desorbed from anti-apoB-100 disk based on their cholesterol analysis with different plasma volumes (220, 320, and 420 µL) of plasma.

1.8 Determination of suitable flow rate for the isolation of LDL

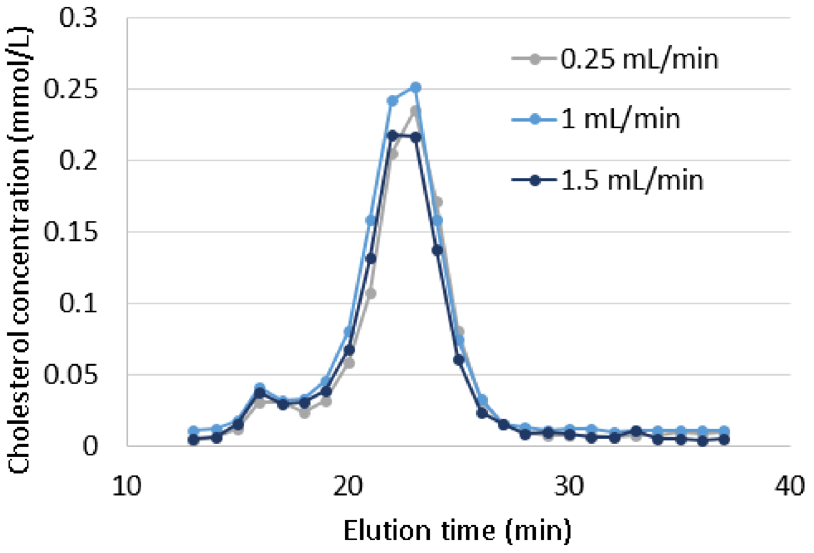

Figure S12. FPLC profiles of LDL particles desorbed from anti-apoB-100 disk based on their cholesterol analysis. Different flow rates (0.25 mL/min, 1.0 mL/min, and 1.5 mL/min) used in the anti-apoB-100 disk are displayed.

Supplementary section 2: Isolation and characterization of isolates from C6S and anti-apoB-100 disks

2.1 ELISA results

Table S4. ApoB-100 and apoE concentrations measured by ELISAs.

**Sample**	**apoB-100 (µg/mL)**	**apoE (µg/mL)**
Fraction 1	481	6.35
Fraction 2	582	1.53
Fraction 3	118	0
Fraction 4	50.9	0
Combined fractions	1232	7.88
Original plasma	1355	30.64
Recovery	91%	25.7%

2.2 FPLC chromatograms of fractionated isolates from C6S and anti-apoB-100 disks

(a) Isolate from C6S disk

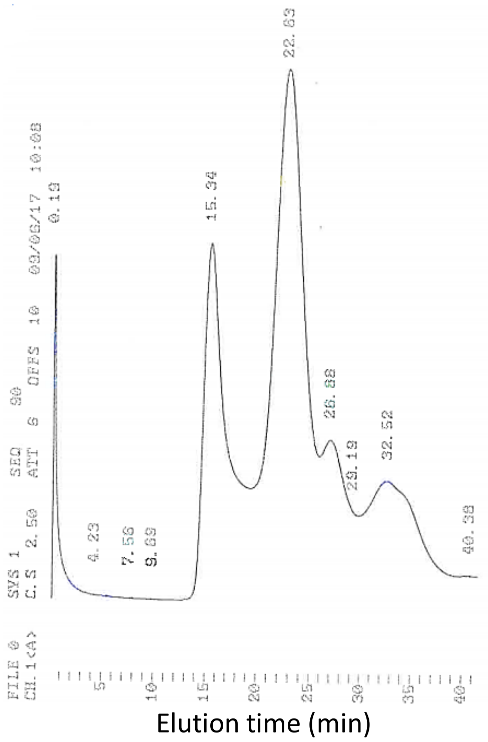

Peak areas (detection at 280 nm)

FILE : 0
CALC-METHOD: AR/HI% <AREA> COMPONENT

NO.	RT	AREA	CONC	BC
1	0.19	1009184	4.199	BV
2	4.23	443	0.002	TBB
3	4.82	444	0.002	TBB
4	5.41	359	0.001	TBB
5	7.56	191	0.001	TBB
6	7.78	787	0.003	TBB
7	9.69	530	0.002	TBB
8	11.27	248	0.001	BB
9	15.34	5879732	24.467	BV
10	22.63	13324774	55.447	VV
11	26.88	361339	1.504	TBB
13	32.52	3449054	14.352	VV
14	40.38	4498	0.019	TBB
TOTAL		24031563	100.000	

PEAK REJ : 0

(b) Isolate from anti-apoB-100 disk

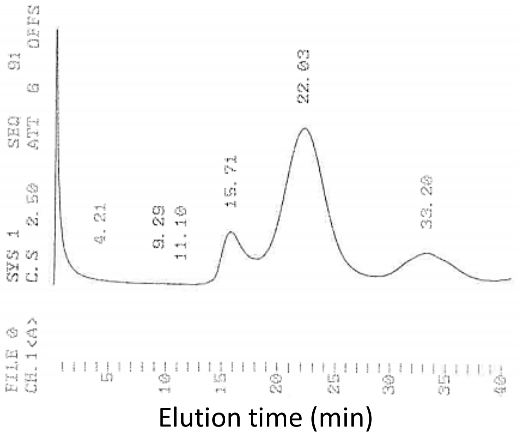

Peak areas (detection at 280 nm)

FILE : 0
CALC-METHOD: AR/HI% <AREA> COMPONENT

NO.	RT	AREA	CONC	BC
1	0.19	749258	12.385	BV
2	4.21	527	0.009	TBB
3	4.80	251	0.004	TBB
4	9.29	135	0.002	TBB
5	9.52	242	0.004	TBB
6	9.84	108	0.002	BB
7	10.89	216	0.004	BB
8	11.10	372	0.006	BB
9	11.50	236	0.004	BB
10	11.87	91	0.002	BB
11	15.71	725996	12.000	BV
12	22.03	3757580	62.111	VV
13	33.20	814738	13.467	VB
TOTAL		6049752	100.000	

PEAK REJ : 0

Figure S13. FPLC chromatograms and peak areas of fractionated lipoprotein particles from (a) C6S disk and (b) anti-apoB-100 disk. The plasma sample injected to the anti-apoB-100 disk was 320 µL diluted to the total volume of 5 mL with PBS. The eluent was carbonate-bicarbonate solution (0.1 M, pH 11.3). The experiments were performed in duplicate, and the isolates were combined. The FPLC and isolation flow rate for the disks was 0.5 mL/min. FPLC detection was done at $A_{280\ nm}$, and (c) total protein profiles of fractionated lipoprotein particles from the anti-apoB-100 disk (red) and C6S disk (blue).

(c) Total protein profiles

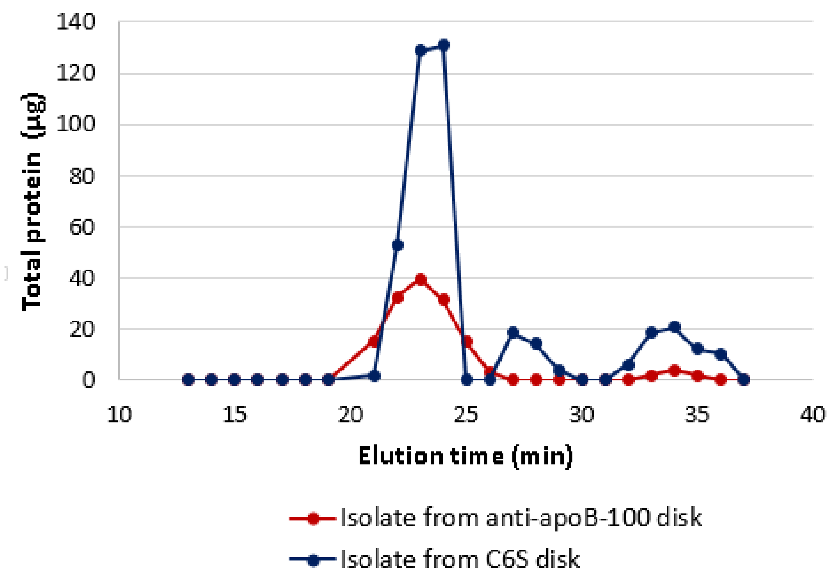

Figure S13 (continued). FPLC chromatograms and peak areas of fractionated lipoprotein particles from (a) C6S disk and (b) anti-apoB-100 disk. The plasma sample injected to the anti-apoB-100 disk was 320 µL diluted to the total volume of 5 mL with PBS. The eluent was carbonate-bicarbonate solution (0.1 M, pH 11.3). The experiments were performed in duplicate, and the isolates were combined. The FPLC and isolation flow rate for the disks was 0.5 mL/min. FPLC detection was done at $A_{280\ nm}$, and (c) total protein profiles of fractionated lipoprotein particles from the anti-apoB-100 disk (red) and C6S disk (blue).

2.3 ELISA results

Table S5. Recoveries of total protein and apoB-100 levels in plasma and isolates from the C6S and the anti-apoB-100 disks.

Sample	apoB-100 (µg)	Total protein (µg)	apoB-100 recovery %	Total protein recovery % (apoB-100/ Total protein of disks)
Original plasma	598	N/A		
Isolate from anti-apoB-100 disk	153	152	26 %	100%
Isolate from C6S disk	32.4	420	5.4%	7.7%
Disks total	185.4	572	31 %	96%*
Flow-through plasma	21.3	N/A		

N/A refers to not applicable

Original plasma volume injected was 640 µL.

*Total protein of disks/total apoB-100 of original plasma

2.4 Further studies on the C6S-bound particles using the anti-apoB-100 disk

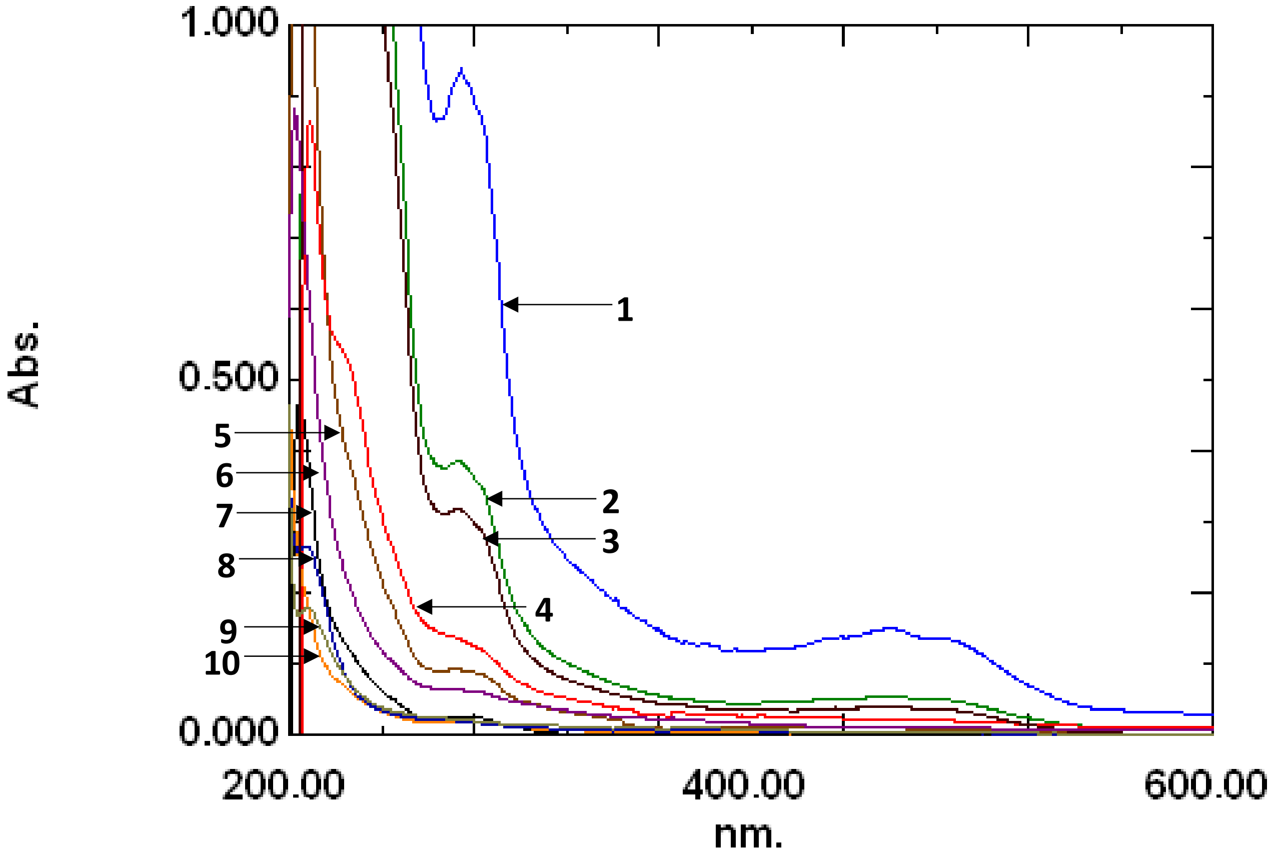

Figure S14. UV-VIS spectra at 200 to 600 nm: (1) ultracentrifugally isolated LDL (400 mg/mL; 200 µg in PBS), (2) isolate from the anti-apoB-100 disk (200 µg of LDL injected), (3) isolate from the C6S disk (200 µg of LDL injected), (4) isolate from the anti-apoB-100 disk ((3) injected), (5) and (6) flow-through (unbound) LDL through the C6S disk, (8) and (9) flow-through LDL through the anti-apoB-100 disk (200 µg of LDL injected), and (7) and (10) flow-through LDL through the anti-apoB-100 disk ((3) injected).

2.5 QCM analysis

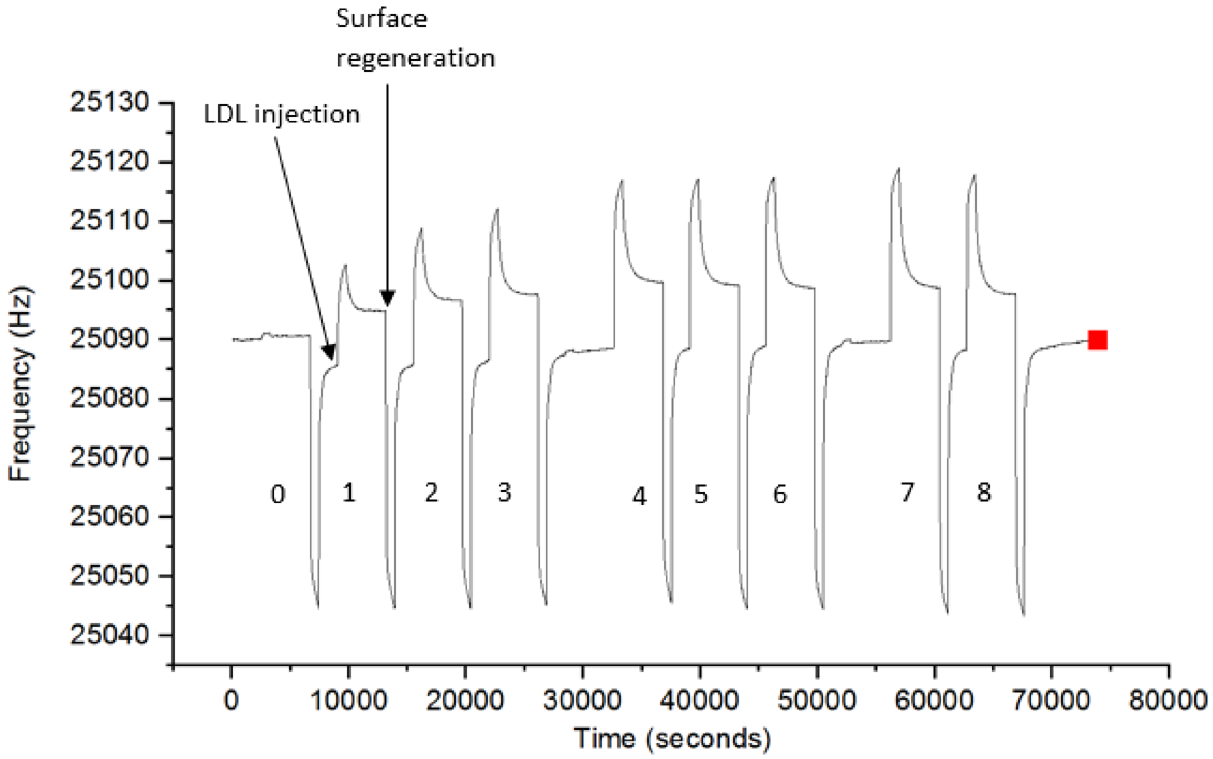

Figure S15. Representative QCM responses for the LDL interactions with the anti-apoB-100 mAb at 37°C. Serial injections of different LDL concentrations were done on the sensor chip immobilized with the anti-apoB-100 mAb. Numbers under each measurement indicate LDL concentration: 1. 10 nM (5 µg/mL), 2. 29 nM (15 µg/mL), 3. 59 nM (30 µg/mL), 4. 117 nM (60 µg/mL), 5. 176 nM (90 µg/mL), 6. 234 nM (120 µg/mL), 7. 293 nM (150 µg/mL), and 8. 352 nM (180 µg/mL), while 0 refers to blank injection (0 nM LDL). LDL concentrations were based on its total protein content. Surface regeneration was achieved by NH_4OH injections (0.28 M, 90 µL).

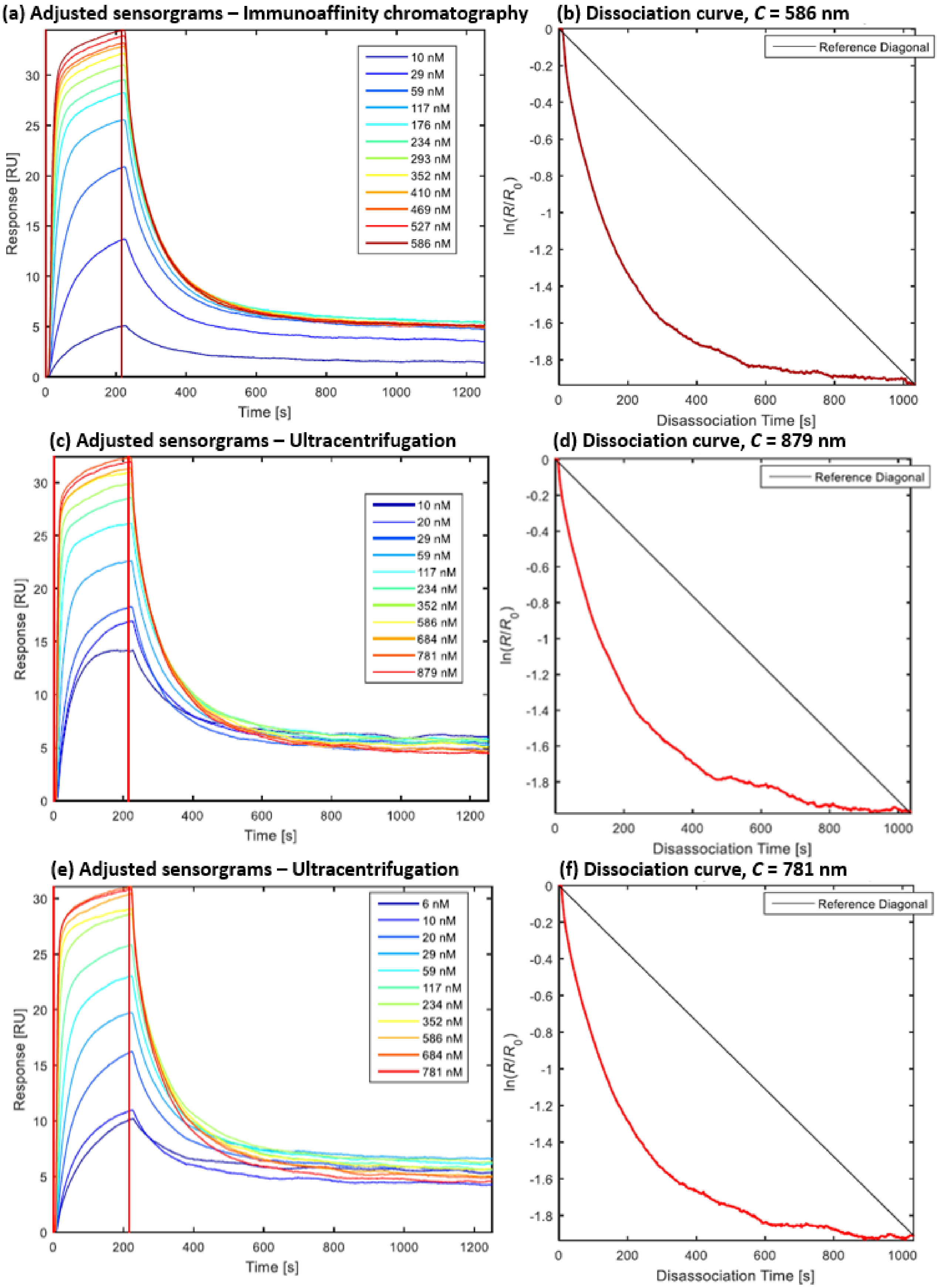

Figure S16. Adjusted sensorgrams for interactions of LDL isolated from plasma of individual#1 using affinity chromatography method (a)-(b) and ultracentrifugation (c)-(f) with the anti-apoB-100 mAb and their corresponding disassociation graphs. The injection end is indicated as a vertical red line.

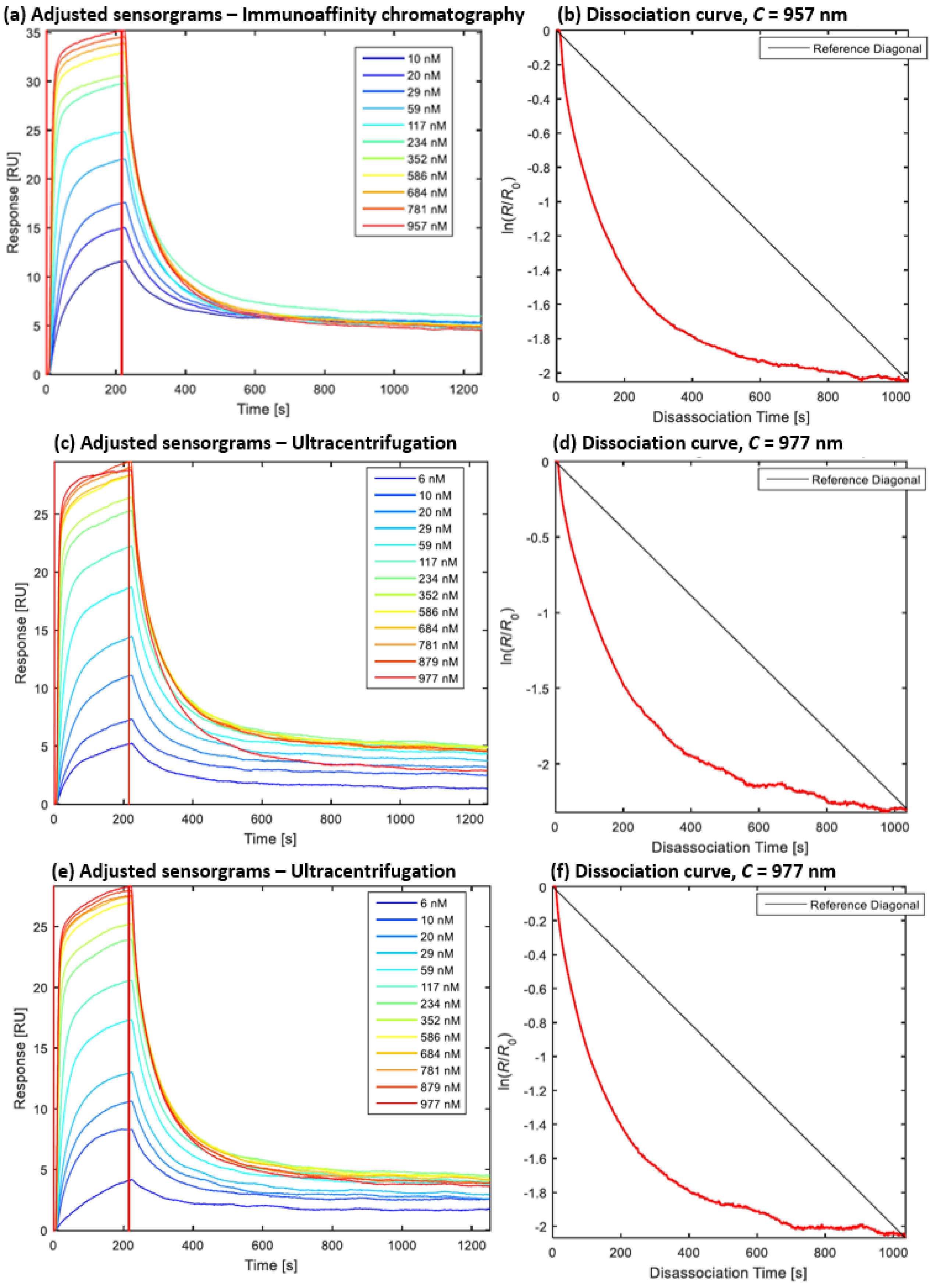

Figure S17. Adjusted sensorgrams for interactions of LDL isolated from plasma of individual#2 using our affinity chromatography method (a)-(b) and ultracentrifugation (c)-(f) with the anti-apoB-100 mAb and their corresponding disassociation graphs. The end of injection is indicated as a vertical red line.

Table S6. Median rate constants and dissociation equilibrium constants with 95% confidence intervals of anti-apoB-100 mAb and LDL isolated with monolithic disk system and ultracentrifugation. Data analysis was done with AIDA.

Interaction	Individual	Isolation method	$\log(k_a)$ [$M^{-1}s^{-1}$]	$\log(k_d)$ [s^{-1}]	$\log K_D$ [M]
1	1	Affinity chromatography	5.38	-4.20	-9.58
		Ultracentrifugation	5.75	-5.31	-11.06
		Ultracentrifugation	5.83	-4.85	-10.68
	2	Affinity chromatography	5.70	-3.67	-9.37
		Ultracentrifugation	5.50	-3.64	-9.14
		Ultracentrifugation	5.46	-4.58	-10.04
2	1	Affinity chromatography	3.79	-4.85	-8.64
		Ultracentrifugation	4.29	-5.26	-9.55
		Ultracentrifugation	4.06	-4.88	-8.94
	2	Affinity chromatography	3.67	-4.71	-8.38
		Ultracentrifugation	3.83	-3.87	-7.70
		Ultracentrifugation	3.92	-4.64	-8.56
3	1	Affinity chromatography	3.50	-2.31	-5.81
		Ultracentrifugation	3.96	-2.26	-6.22
		Ultracentrifugation	4.17	-2.11	-6.28
	2	Affinity chromatography	3.75	-2.21	-5.96
		Ultracentrifugation	3.92	-2.11	-6.03
		Ultracentrifugation	3.83	-2.21	-6.04
4	1	Affinity chromatography	5.39	-1.97	-7.36
		Ultracentrifugation	5.67	-2.01	-7.68
		Ultracentrifugation	5.79	-2.01	-7.80
	2	Affinity chromatography	5.48	-1.98	-7.46
		Ultracentrifugation	5.73	-1.88	-7.61
		Ultracentrifugation	5.60	-1.92	-7.52
5	1	Affinity chromatography	5.00	-1.14	-6.14
		Ultracentrifugation	5.29	-0.94	-6.23
		Ultracentrifugation	5.25	-0.85	-6.10
	2	Affinity chromatography	5.08	-1.19	-6.27
		Ultracentrifugation	5.29	-0.92	-6.21
		Ultracentrifugation	5.13	-1.04	-6.17

Supplementary section 3: Methods

3.1 Chemicals and materials

1-Ethyl-3-(3-dimethylaminopropyl) carbodiimide hydrochloride (EDC-HCl, 98.5%) and sulfo-*N*-hydroxy-succinimide (S-NHS, 98.0%) were purchased from Aladdin Chemical Reagent Company (Shanghai, China). 4-(2-Hydroxyethyl)-1-piperazineethanesulfonic acid (HEPES), HCl (fuming 37%), KBr, ethanolamine, TWEEN® 20, acetone, and chondroitin-6-sulfate (C6S) were purchased from Sigma-Aldrich (St. Louis, MO, USA). Ammonia (25%) was purchased from Riedel-de Haën (Seelze, Germany). Phosphate buffered saline (PBS) tablets were purchased from Sigma-Aldrich (St. Louis, MO, USA). NaCl was purchased from ThermoFisher Scientific. NaOH (1.0 M) and HCl (1.0 M) were purchased from Oy FF Chemical Ab (Yli-li, Finland). $NaHCO_3$, Na_2CO_3, and acetic acid were purchased from Merck KGaA (Darmstadt, Germany). Orthophosphoric acid (85%) was purchased form VWR Chemicals. Water was obtained with MilliQ system (Millipore, USA). Bovine serum albumin (BSA) was purchased from Sigma Aldrich (St. Louis, MO, USA).

3.2 Instrumentation

CIM® carbonyldiimidazole (CDI) disks (0.34 mL, pore size 1.3 µm) used as stationary phases for affinity separations and housing cartridges were provided by BIA Separation (Ljubljana, Slovenia). The Quartz Crystal Microbalance (QCM) instrument (Attana AB, Stockholm, Sweden) (Model A100) was used to analyse interactions of anti-apoB-100 mAb with LDL using Attana LNB-Carboxyl sensor chips (Attana, Stockholm, Sweden). FPLC system (Merck Hitachi Ltd., Tokyo, Japan) for the analysis of biological samples consisted of a pump model L-6200A, a UV-vis detector model L-4200, an integrator model D-7500, and the column, Superose® 6 HR 10/300 column lot 17-5172-01 (GE Healthcare Bio-Sciences Ab, Uppsala, Sweden). The syringe pump (SP100i, World Precision Instruments Inc., USA) was used for sample introduction to the monolithic columns. Human apolipoprotein B-100 ELISA^{PRO} kit (code 3715-1HP-2) was purchased from Mabtech AB, Sweden. Roche Cholesterol CHOD-PAP reagent (kit no. 1489232) for quantification of total cholesterol was obtained from Roche, Germany. Bio-Rad DC™ Protein Assay kit for total protein analysis was purchased from Bio-Rad Laboratories (Hercules, CA). The absorptions were measured using an EnSpire® 2300 Multilabel Plate Reader (PerkinElmer Inc., USA). UV-vis detectors (Models SPD-20AV Prominence

and UV-1800, Shimadzu) were used for measuring absorptions during the affinity chromatography method development. Millipore filters (pore size 0.45 µm) (Merck Millipore Ltd.) were used to filter solutions. The centrifuge Heraeus Biofuge Pico (ThermoFisher Scientific) was used for plasma and lipoprotein treatments. Isolation of lipoproteins from plasma was performed using Beckman Optima TL Table-Top ultracentrifuge (Beckman Coulter, Brea, CA).

3.3 Preparation of solutions

PBS solution (I = 10 mM, 2.7 mM KCl and 137 mM NaCl, pH 7.4) was prepared by dissolving a PBS tablet in 200 mL of MilliQ water. PBS used during the immobilization was filtered through 0.45 µm filters. NH_4OH (0.28 M, pH 11.5) used as an eluent for affinity chromatography and a regeneration solution for QCM experiments was prepared by diluting 1.13 mL of 25% ammonia solution with MilliQ water to the final volume of 50 mL. C6S solution (5 mg/mL, 5 mL) for affinity monolith chromatography was prepared by dissolving 25 mg of C6S powder in 5 mL of PBS to obtain the final concentration of 5 mg/mL and filtered through a 0.45 µm filter. Anti-apoB-100 solution (0.5 mg/mL, 2.5 mg) for immunoaffinity chromatography was prepared by diluting 480 µL of anti-human-apolipoprotein B-100 monoclonal antibody (anti-apoB-100 mAb) (5.2 mg/mL) provided by Medix Biochemica Co. Inc. (Helsinki, Finland) to the final volume of 5 mL with PBS. Ethanolamine solution (2 M, pH 9.0) was prepared by diluting 0.60 mL of ethanolamine with 5 mL of MilliQ water. pH of the solution was adjusted with 900 µL of HCl (from HCl fuming 37%) to pH 9.0. Sodium carbonate-bicarbonate solution (0.1 M, pH 11.3) used as an eluent for immunoaffinity chromatography was prepared by mixing 90 mL of 0.1 M Na_2CO_3 solution with 10 mL of 0.1 M sodium $NaHCO_3$ solution. pH of the solution was adjusted to 11.3 with 1 M NaOH.

QCM running buffer during immobilization, 4-(2-hydroxyethyl)-1-piperazineethanesulfonic acid) (HEPES) (pH 7.4, I = 10 mM, 150 mM NaCl, 0.005% TWEEN 20), was prepared by dissolving 1.19 g of HEPES and 4.38 g of NaCl in 400 mL of MilliQ water. The mixture was added with 0.25 mL of 10% TWEEN 20, and the pH was adjusted to 7.4 with 1 M NaOH. EDC-HCl solution (0.4 M before mixing, 1 mL) was prepared by dissolving 76.7 mg of EDC-HCl in 1 mL of MilliQ water. S-NHS solution (0.1 M before mixing, 1 mL) was prepared by dissolving 21.7 mg of S-NHS in 1 mL of MilliQ water. Deactivation solution, ethanolamine (1 M, pH 9.0), was prepared by diluting 500 µL of 2 M ethanolamine with 500 µL of MilliQ water. Anti-apoB-100

mAb solution (binding ligand solution) (0.1 mg/mL, 1 mL) was prepared by mixing 19.2 µL of anti-apoB-100 mAb (5.2 mg/mL) in 1 mL of pH adjusted PBS (pH 6.4). C6S solution (binding ligand solution for preliminary affinity ligand selection) (100 mg/mL, 1 mL) was prepared by dissolving 100 mg of C6S in 1 mL of acetate buffer (I = 10 mM, pH 4.0). The acetate buffer (I = 10 mM, pH 4.0) was prepared by diluting 57 µL of acetic acid in 50 mL of MilliQ water. The pH of solution was adjusted with 1.0 M NaOH. The immobilization solution for C6S ligand was HEPES prepared in similar manner described above without the addition of TWEEN 20. The binding solution for C6S ligand was phosphate buffer (I = 25 mM, 25 mM NaCl, pH 7.4) prepared by dissolving 85% phosphoric acid (485 µL) in 1 M NaOH (14.2 mL) and MilliQ water (1 L). NaCl (1.46 g) was added to the mixture, and the pH of the solution was adjusted with 1 M HCl.

3.4 Preparation of biological samples

Human LDL (d = 1.019-1.050 g/mL) was isolated from non-fasting plasma using sequential ultracentrifugation as described in [1]. Briefly, EDTA (final concentration 3 mM) and gentamicin sulfate (100 µg/mL) were added to plasma. First, the removal of VLDL and IDL were isolated by adjusting the density of the mixture to 1.019 g/mL by the addition of solid KBr. The mixture was centrifuged at 40,000 rpm (rotor 50.2 Ti, gmax 302 000) at 4°C for 24 h. VLDL and IDL were removed from the top, and the density of the remaining plasma was adjusted to 1.050 g/mL with solid KBr. The mixture was centrifuged at 40,000 rpm for 72 h. The top fraction containing LDL was collected and centrifuged for another 24 h at the density of 1.063 g/mL. LDL collected from the top was dialyzed extensively against 1 mM EDTA-150 mM NaCl (pH 7.4). The concentration of the isolated LDL was measured using a BCA protein assay kit (Pierce, Rockford, IL). The VLDL-LDL mixture, containing chylomicrons, VLDL, and VLDL remnants including IDL, used in this study was isolated from plasma in a similar manner, but the density-adjusted plasma (d = 1.019 g/mL) was centrifuged for 2 h at 6°C at 100,000 rpm using the Table-Top ultracentrifuge (Beckmann Optima TL, USA) (rotor TLA-100.3, 1). The VLDL-IDL mixture (1 mL) was recovered from top part of the centrifugal tube (1 mL). Human blood plasma samples were obtained from non-fasting healthy individuals and provided by the Finnish Red Cross Blood Service (Helsinki, Finland). These plasma samples were used in the affinity chromatography method development, and they were centrifuged for 5 min at 13,000 rpm using Heraeus Biofuge Pico centrifuge (ThermoFisher Scientific) to remove protein aggregates. The LDL samples used in QCM experiments were

isolated from fresh plasma samples obtained from the Finnish Red Cross Blood Service with the isolation protocol described above to avoid particle aggregations. LDL solutions having different concentrations were obtained by diluting with PBS (pH 7.4). Human monoclonal anti-human-apolipoprotein B-100 (5.2 mg/mL, code Anti-h ApoB 2101 SPTN-5) was provided by Medix Biochemica Co. Inc. (Helsinki, Finland). The production of anti-apoB-100 mAb was described in [1].

3.5 Development of the isolation method.

Affinity ligand selection. To select the suitable affinity ligands, preliminary QCM studies were conducted to evaluate binding behaviours between the ligand (C6S) and the analyte (VLDL-IDL mixture). All of the following steps were performed with an Attana A100 QCM biosensor instrument (Attana AB, Sweden). The immobilization of C6S on an LNB-carboxyl sensor chip (Attana, Stockholm, Sweden) was done with amine coupling procedure. Prior to the immobilization, the LNB-Carboxyl sensor chip was pre-wetted *ex-situ* with 20 µL of MilliQ water, inserted to the instrument, and left to stabilize. The immobilization was performed with the immobilization buffer (10 mM HEPES, 150 mM NaCl, pH 7.4) at the flow rate of 10 µL/min at 25°C. The surface activation was done by three injections of 0.4 M EDC and 0.1 M S-NHS (1:1, v/v) for 400 s each, using the automated C-Fast software. The amine coupling of C6S (100 mg/mL) was carried out by injecting the solution twice for 200 s each. Finally, the remaining activated carboxyl groups were deactivated with three injections of ethanolamine solution (1 M, pH 9.0) for 400 s each. The running buffer was changed to phosphate buffer (I = 20 mM, 25 mM NaCl, pH 7.4). Thereafter, the VLDL-IDL mixture containing chylomicrons, VLDL, VLDL remnants, including IDL particles (35 µL) having concentrations ranging from 10.24 mg/mL to 200 mg/mL (20 nM to 391 nM) were injected to the chip at a flow rate of 50 µL/min. Molar concentrations of VLDL and IDL were calculated using molecular weight of one of their apolipoproteins, apoB-100 (512 kDa). The regeneration was performed with NH_4OH (0.28 M, pH 11.5).

Desorption experiments. Suitable eluents for both C6S and anti-apoB-100 disks were determined using a variety of buffers and solutions with different properties presented in Supplementary Tables S1 and S2. The desorption profile of the C6S disk was obtained by the injection of 200 µL of the ultracentrifugally separated VLDL-IDL mixture diluted to 5 mL with PBS (pH 7.4) to the C6S disk using a syringe pump, and the desorption was done with NH_4OH (0.28 M, pH 11.5). The isolate was subjected to FPLC. Initial experiments

with the anti-apoB-100 were performed by injecting Dil-labeled LDL to the anti-apoB-100 disk. The success of desorption was determined by the colour change of the disk surface before and after desorption as well as the presence of orange-red colour in the eluate. In addition, desorption profiles of the anti-apoB-100 disk (both before and after the deactivation of active CDI groups) were obtained by the injection of 1 mL of plasma, followed by injections of PBS (2 mL, pH 7.4) and NH_4OH (2 mL, 0.28 M, pH 11.5), and monitored using a UV detector (SPD-20AV Prominence, Shimadzu). Additional experiments were done by injecting 400 µL of plasma diluted to 5 mL with PBS (pH 7.4) to the anti-apoB-100 disk in order to find the most optimal eluent apart from the NH_4OH (0.28 M, pH 11.5). The selected eluent to be tested was carbonate-bicarbonate solution (0.1 M, pH 11.3). Then, the disk was washed with NH_4OH (0.28 M, pH 11.5). Eluates from both solutions were collected and injected to the FPLC system.

Studies of CDI disk and non-specific adsorption. To test the effect of the deactivation of active CDI groups on the monolith surface to non-specific adsorption of particles from plasma, plasma samples (600 µL of plasma diluted to the total volume of 1.2 mL with PBS) were injected to a CDI monolithic disk (without immobilized affinity ligands) both before and after the deactivation with ethanolamine (2 M, pH 9.0). The desorption was done with NH_4OH (0.28 M, pH 11.5) with the flow rate of 0.25 mL/min. Similar experiments were performed with the anti-apoB-100 disk before and after deactivation with the injection of 400 µL of plasma diluted to the total volume of 5 mL with PBS. The desorption was done with carbonate-bicarbonate solution (0.1 M, pH 11.3). Isolates from these experiments were injected to the FPLC system for fractionation. Total cholesterol levels in fractionated isolates of the anti-apoB-100 disks were measured.

Dynamic binding capacity. To assess the dynamic binding capacity of the anti-apoB-100 disk before and after deactivation of the active CDI groups, frontal analysis experiments were performed as described in [2]. Different LDL concentrations ranging from 175 µg/mL to 450 µg/mL were injected to deactivated and non-deactivated anti-apoB-100 disks. The LDL concentrations were based on the measured total protein concentration. The system void volume was measured using BSA, and breakthrough curves were monitored through the UV detector at 280 nm. The desorption of bound particles on the anti-apoB-100 disk was performed with NH_4OH (0.28 M, pH 11.5).

Effect of flow rate and plasma injection volumes. Three different plasma volumes, 220, 320, and 420 µL diluted to 5 mL in PBS (pH, 7.4) were injected to the two-disk system containing C6S and anti-apoB-100 disks arranged in tandem to determine the optimal plasma volume for the monolithic disk system. The operation flow rate was 0.5 mL/min. The desorption was done with NH_4OH (0.28 M, pH 11.5). Different flow rates ranging from 0.25 to 1.5 mL/min were also tested with the anti-apoB-100 disk to determine their effects on LDL binding. The original plasma was 400 µL of plasma diluted to 5 mL with PBS. The bound particles were desorped with carbonate-bicarbonate solution (0.1 M, pH 11.3). The most suitable flow rate and plasma injection volume were assessed based on cholesterol levels measured in each fraction obtained from fast protein liquid chromatography (FPLC).

3.6 Binding experiment of the isolate from C6S disk

To study the binding properties of the isolate from the C6S disk to anti-apoB-100 mAb, two additional experiments were conducted. First, ultracentrifugally purified LDL (200 µg diluted to 500 µL with PBS, pH 7.4) was introduced to the C6S disk. Bound fraction from the C6S disk was desorbed using NH_4OH (0.28 M, pH 11.5), neutralized, and introduced to the anti-apoB-100 disk. The absorbance of LDL and isolates from both disks at 200 to 600 nm was measured using a UV-vis spectrophotometer (UV-1800, Shimadzu). Second, we introduced ultracentrifugally purified LDL (200 µg diluted to 500 µL with PBS, pH 7.4) to the anti-apoB-100 disk. The desorption and absorbance measurement were conducted in the same way described above.

References

1. Multia, E. *et al.*, Thermodynamic and kinetic approaches for evaluation of monoclonal antibody-Lipoprotein interactions. *Anal. biochem.* **518**, 25-34 (2017).
2. Hage, D.S. & Cazes, J., *Handbook of Affinity Chromatography* (Second Edition) 613-616 (Taylor & Francis, 2005).
